# New insights into the NH_3_-selective catalytic reduction of NO over Cu-ZSM-5 as revealed by *operando* spectroscopy†

**DOI:** 10.1039/d1cy02348a

**Published:** 2022-02-28

**Authors:** Xinwei Ye, Ramon Oord, Matteo Monai, Joel E. Schmidt, Tiehong Chen, Florian Meirer, Bert M. Weckhuysen

**Affiliations:** Institute of New Catalytic Materials Science, School of Materials Science and Engineering, Key Laboratory of Advanced Energy Materials Chemistry (MOE), Nankai University Tianjin 300350 China; Inorganic Chemistry and Catalysis Group, Debye Institute for Nanomaterials Science, Utrecht University Universiteitsweg 99 3584 CG Utrecht The Netherlands b.m.weckhuysen@uu.nl

## Abstract

To control diesel vehicle NO_*x*_ emissions, Cu-exchanged zeolites have been applied in the selective catalytic reduction (SCR) of NO using NH_3_ as reductant. However, the harsh hydrothermal environment of tailpipe conditions causes irreversible catalyst deactivation. The aggregation of isolated Cu^2+^ brings about unselective ammonia oxidation along with the main NH_3_-SCR reaction. An unusual ‘dip’ shaped NO conversion curve was observed in the steamed zeolite Cu-ZSM-5, resulting from the undesired NH_3_ oxidation that produced NO. Here we gain further insights into the NH_3_-SCR reaction and its deactivation by employing *operando* UV-vis diffuse reflectance spectroscopy (DRS) and diffuse reflectance infrared Fourier transform spectroscopy (DRIFTS) on fresh and steamed zeolite Cu-ZSM-5. We found that tetragonally distorted octahedral Cu^2+^ with associated NH_3_ preferentially forms during low temperature NH_3_-SCR (<250 °C) in fresh Cu-ZSM-5. The high coordination number of Cu^2+^ ensures the availability for high coverage of nitrate intermediates. Whilst in the steamed Cu-ZSM-5, [Cu_*x*_(OH)_2*x*−1_]^+^ oligomers/clusters in pseudo-tetrahedral symmetry with coordinated NH_3_ accumulated during the low-temperature NH_3_-SCR reaction. These clusters presented a strong adsorption of surface NH_3_ and nitrates/nitric acid at low temperatures and therefore limited the reaction between surface species in the steamed Cu-ZSM-5. Further release of NH_3_ with increased reaction temperature favors NH_3_ oxidation that causes the drop of NO conversion at ∼275 °C. Moreover, competitive adsorption of NH_3_ and nitrates/nitric acid occurs on shared Lewis-acidic adsorption sites. Prompt removal of surface nitrates/nitric acid by NO avoids the surface blockage and tunes the selectivity by alternating nitrate–nitrite equilibrium. The formation of adsorbed NO_2_ and HNO_*x*_ points to the necessity of an acid adsorbent in practical applications. The structural similarity under the NH_3_-SCR reaction and unselective NH_3_ oxidation confirmed the entanglement of these two reactions above 250 °C.

## Abstract 2

To control diesel vehicle NO_*x*_ emissions, Cu-exchanged zeolites have been applied in the selective catalytic reduction of NO using NH_3_ as reductant. In this work, the local structure of Cu^2+^ in zeolite ZSM-5 has been revealed by *operando* spectroscopies.

## Graphical abstract

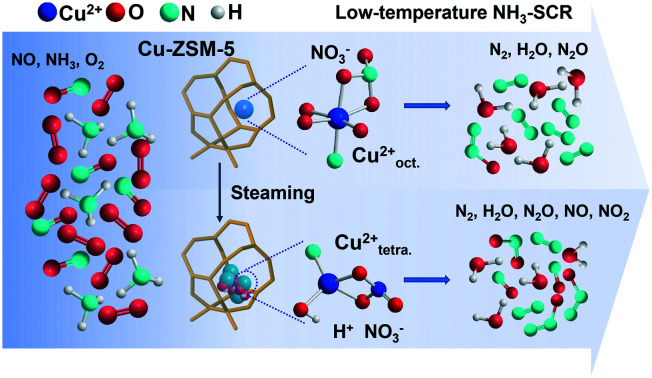

## 1. Introduction

Emission control of NO_*x*_ (*i.e.*, NO, N_2_O and NO_2_) has been mandated in applications such as stationary power plants and diesel engine vehicles. Vanadia-based NH_3_-selective catalytic reduction (NH_3_-SCR) catalysts are rather efficient and economical in stationary NO_*x*_ abatement, but failed to adapt to diesel vehicles because of the low activity at a high air/fuel-ratio and their high SO_2_ oxidation activity.^1,2^ Considering the dominant emission of NO compared to N_2_O and NO_2_ in NO_*x*_-lean automotive exhausts, the standard NH_3_-SCR reaction (4NH_3_ + 4NO + O_2_ = 4N_2_ + 6H_2_O), where stoichiometrically equal amounts of NH_3_ as NO are employed, is the main focus in catalyst development.^3^ Catalysing such a redox reaction, involving electron transfer processes, requires a catalyst that can accept and donate electrons when encountering reactant molecules or bind with reaction intermediates. Transition metal-based catalysts are thus promising candidates for NH_3_-SCR, due to the modifiable electron configuration of their d orbitals.

Since the high NO decomposition activity of zeolite Cu-ZSM-5 was discovered in 1980s, Cu-exchanged zeolites have been widely investigated for the NH_3_-SCR reaction.^4^ Although Cu-exchanged zeolites exhibit high NH_3_-SCR activity over a wide temperature window, the automotive industry is still facing the dilemma of choosing a suitable catalyst for commercialization – medium/large pore zeolite structures, such as MFI and BEA, are limited by their low hydrothermal stability, while the more robust small pore zeolite CHA (*i.e.*, SSZ-13 and SAPO-34) has a higher cost. The irreversible hydrothermal aging of zeolites is a subtle yet permanent process, during which the functional moieties in Cu-exchanged zeolites undergo a dynamic transformation starting from local distortion of the structural unit regardless of the type of zeolite framework. The deactivation of catalysts should be particularly considered for the rational design of emission control systems for vehicle tailpipes.

The ideal Cu species in Cu-exchanged zeolites are isolated Cu^2+^ balanced by an Al pair and [CuOH]^+^ balance by a single Al site. When the Cu-exchanged zeolites undergo hydrothermal treatment or experience a deactivation process, the degradation of Cu increases the heterogeneity of Cu species. The Cu_*x*_O_*y*_ clusters/nanoparticles, spinel phase CuAl_2_O_4_, as well as Cu(OH)_2_ can form and are considered to be detrimental for the standard NH_3_-SCR reaction.^5–9^ Various Cu species in the zeolites provide multiple possible sites for catalytic reactions at NH_3_-SCR reaction conditions. Undesired byproducts, for instance NO_2_ and N_2_O, can be selectively formed during the NH_3_-SCR reaction.^10^ Additionally, with multiple evolutionary Cu species in the Cu-exchanged zeolites, the unwanted side reactions such as NO oxidation (2NO + O_2_ = 2NO_2_) and unselective NH_3_ oxidation to NO (4NH_3_ + 5O_2_ = 4NO + 6H_2_O) can also take place under standard NH_3_-SCR reaction conditions.^11–14^

In our previous study of steamed Cu-ZSM-5 zeolites we have observed an unusual NO conversion curve with a ‘dip’ shape at around 300 °C.^15^ A similar drop of NO conversion was reported at ∼270 °C with the hydrothermally treated zeolite Cu-SSZ-13 and was simply explained by the accelerated unselective NH_3_ oxidation promoted by Cu_*x*_O_*y*_ clusters/nanoparticles.^16,17^ However, the detailed structural reasons for the low NH_3_-SCR activity have not yet been well understood due to the interference of multiple Cu sites and side reactions.

**Fig. 1:**
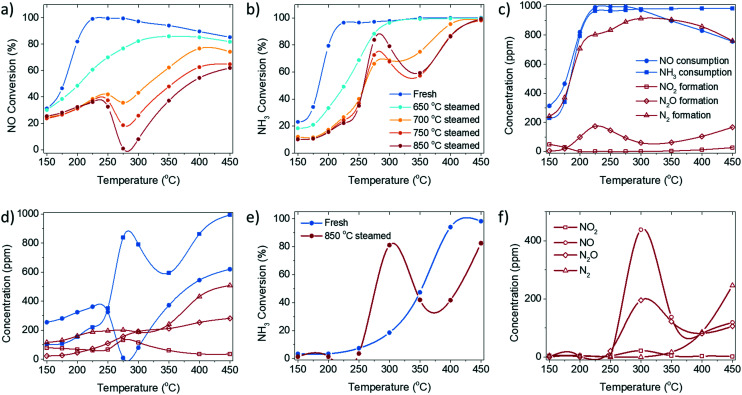
a) NO conversion and b) NH_3_ conversion of the standard NH_3_-selective catalytic reduction (SCR) reaction performed over fresh and steamed Cu-ZSM-5. The reactant consumption and product formation in standard NH_3_-SCR on c) fresh and d) 850 °C steamed zeolite Cu-ZSM-5. e) The conversion of NH_3_ during the NH_3_ oxidation reaction over the fresh and steamed Cu-exchanged ZSM-5. f) Product concentration from the NH_3_ oxidation reaction over 850 °C steamed Cu-ZSM-5. The standard NH_3_-SCR reaction was conducted with a gas hourly space velocity (GHSV) of 100 000 h^−1^ with 1000 ppm NO, 1000 ppm NH_3_, 5% O_2_ and balanced with He. The NH_3_ oxidation reaction was conducted with a GHSV of 100 000 h^−1^ with 1000 ppm NH_3_, 5% O_2_ and balanced with He.

In this study, the catalytic performance and structural properties of a series of fresh and steamed Cu-ZSM-5 zeolites were investigated for a more complete understanding of NH_3_-SCR catalysis utilizing Cu-exchanged zeolites by mimicking different aging severities. *Operando* UV-vis diffuse reflectance spectroscopy (DRS) and diffuse reflectance infrared Fourier transform spectroscopy (DRIFTS) were conducted to gain mechanistic insight into the NH_3_-SCR reaction and its deactivation, and to gain a deeper understanding of the unusual catalytic behaviour of the steamed zeolite Cu-ZSM-5 material. The dynamic of structural changes of the Cu^2+^ site under reaction conditions were followed by *operando* UV-vis DRS, specifically by interpretation of the ligand-to-metal charge transfer (LMCT) as well as the d–d transition bands based on crystal field theory. The behaviour of adsorbed species, including chemisorbed NH_3_ and nitrates/nitric acid, were investigated utilizing their development and consumption under various reaction conditions. Finally, the ‘dip’-shaped NO conversion curve (Fig. 1) could be explained by the side reaction of unselective NH_3_ oxidation, which is structurally ascribed to the possible formation of [Cu_*x*_(OH)_2*x*−1_]^+^ oligomers/clusters with a pseudo-tetrahedral Cu^2+^ center, coordinated with NH_3_ in the steamed Cu-ZSM-5 material. The slow rate of surface reaction between adsorbed NH_3_ and surface nitrites/nitrates or nitrous/nitric acid limits the low-temperature NH_3_-SCR.

## 2. Results and discussion

### 2.1 NH_3_-Selective catalytic reduction, NO oxidation and NH_3_ oxidation

The fresh zeolite Cu-ZSM-5 underwent a steaming pre-treatment to simulate the working catalysts after various degrees of deactivation. The standard NH_3_-SCR reaction was performed on fresh and steamed Cu-ZSM-5 catalysts in the temperature range from 150 to 450 °C (Fig. 1, S1 and S2†). A varying extent of deactivation of NH_3_-SCR activity was observed in the steamed zeolites Cu-ZSM-5. The fresh Cu-ZSM-5 showed the highest NO conversion and N_2_ selectivity in the whole temperature range. The steaming process mainly caused the loss of NO conversion at low reaction temperature. It is notable that unusual catalytic behaviour was observed over the zeolites that underwent the 700–850 °C steaming pre-treatment. The NO conversion had a drop starting at 250 °C followed by a continuous increase in conversion from 300 °C, exhibiting a distinct valley-shaped NO conversion curve. Meanwhile, a peak was observed in the NH_3_ conversion curve at the exact temperature of the observed ‘dip’ in NO conversion. This points to the non-equivalent consumption of NO and NH_3_ in the reaction, which contradicts the identical stoichiometric ratio of NO and NH_3_ in a standard NH_3_-SCR reaction.

The conversion and formation of nitrogen-containing compounds in the NH_3_-SCR reaction over fresh and 850 °C steamed Cu-ZSM-5 zeolites are shown in Fig. 1c and d. From the consumption difference between NO and NH_3_, depicted by blue curves, the occurrence of side reactions such as NO or NH_3_ oxidation in standard NH_3_-SCR could be determined. In fresh Cu-ZSM-5, NH_3_-SCR was the favoured reaction, and was only slightly affected by NO oxidation below 200 °C and by NH_3_ oxidation above 300 °C. In contrast, side reactions had more significant impact on the steamed Cu-ZSM-5. This ‘critical temperature’ of 250 °C divides the temperature range into a low- and a high-temperature regime: when the reaction temperature was below 250 °C, the converted NO was overall higher than the converted NH_3_, which implied the involvement of the undesirable NO oxidation, confirmed by the additional production of NO_2_. NH_3_ oxidation hardly contributed to the low temperature regime, proven by no conversion of NH_3_ in the NH_3_ oxidation reaction (Fig. 1e). When the reaction temperature was higher than 250 °C, the consumption of NH_3_ overtook NO consumption, suggesting the involvement of NH_3_ oxidation along with the standard NH_3_-SCR reaction. Especially in the intermediate reaction temperature range of 250–300 °C, the apparent NO conversion dropped to near 0%, while NH_3_ conversion increased, because the NH_3_ oxidation reaction to NO facilitated over the steamed Cu-ZSM-5 (Fig. 1e and f). The produced NO from the NH_3_ oxidation replenished the consumed NO from NH_3_-SCR, and consequently led to the apparent drop in NO conversion from 250 °C in standard NH_3_-SCR (Fig. 1a). In return, the residue NH_3_ was insufficient for the reduction of the surplus NO. As for the N_2_O byproduct, it is formed in the NH_3_-SCR reaction as a partially reduced product of NO through the formation of HNO intermediate.^18^ N_2_O can also be the product of unselective oxidation of NH_3_ (2NH_3_ + 2O_2_ = N_2_O + 3H_2_O). At low reaction temperature, the activity of NH_3_-SCR reaction was high on fresh Cu-ZSM-5, resulting higher N_2_O yield compared to the 850 °C steamed Cu-ZSM-5. With elevated reaction temperatures, the N_2_O generated from both NH_3_-SCR and NH_3_ oxidation reaction kept increasing.

Although the side reaction of NH_3_ oxidation explained the ‘dip’ shape in the NO conversion curve during the NH_3_-SCR reaction, it put forward another puzzle for NH_3_ oxidation conducted over steamed zeolite Cu-ZSM-5, where a peak was observed in the NH_3_ conversion curve at around 300 °C (Fig. 1e). A possible interpretation can be found from a kinetic model of NH_3_ oxidation over Cu-exchanged zeolite Cu-SSZ-13. The reaction at 250–400 °C occurs on Cu-exchanged sites but the NH_3_ conversion decreases with the lower NH_3_ coverage with increasing reaction temperature, while the high temperature reaction (>400 °C) starts to take place on the over-exchanged sites, for instance the Cu_*x*_O_*y*_ species achieving high conversion at elevated temperature.^19^

### 2.2 Changes of the structural properties upon steaming

#### 2.2.1 Local damage of the framework structure

According to the above observed NH_3_-SCR catalytic performance over the fresh and steamed Cu-ZSM-5 zeolites, multiple entangled side reactions present at standard NH_3_-SCR reaction condition, in agreement with the results from previous studies.^8,20,21^ The fresh Cu-ZSM-5 achieved a more stable NO conversion over a wide temperature range, whilst the steamed Cu-ZSM-5 performance was significantly hindered by side reactions throughout the whole temperature range with particularly distinct catalytic performance below 300 °C. Finding the structural reasons behind this complex behaviour was essential for a better understanding of the functions and deactivation of active moieties within Cu-ZSM-5. The zeolite framework is regarded as host for the guest cation through the interaction between the opposite charges of framework O^2−^ and isolated Cu^2+^/[CuOH]^+^. The steaming pretreatment led to loss of zeolite framework crystallinity, revealed by the lower intensity of the diffraction pattern of MFI zeolite (Fig. S4†). This is consistent with the expected partial dealumination due to steaming. It is clearly shown in the Fourier transform infrared (FTIR) spectra in the range of the OH stretching vibration (Fig. S5†) that the well-resolved [CuOH]^+^ (3660 cm^−1^) and Brønsted acid sites (3612 cm^−1^) were replaced by a broad peak representing internal silanol groups in the severely steamed Cu-ZSM-5.^22^ The loss of the Brønsted acid sites upon steaming was an indicator of the formation of local defects within the zeolites, resulting in the heterogeneity of hydroxyl groups, which is experimentally evidenced by the transformation of geometric structure of Al from tetrahedral to octahedral *via* solid-state nuclear magnetic resonance (ssNMR) and X-ray absorption spectroscopy (XAS).^22,23^

#### 2.2.2 Loss of isolated Cu^2+^ sites

**Fig. 2:**
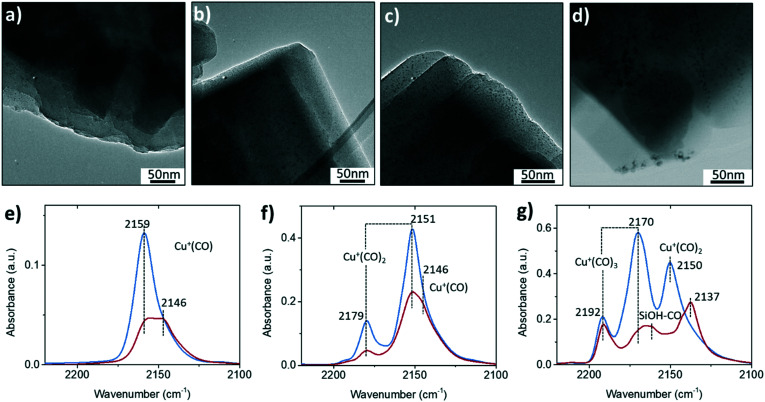
Transmission electron microscopy (TEM) images of a) fresh, b) 650 °C steamed and c–d) 850 °C steamed zeolite Cu-ZSM-5 in bright field. The CO-adsorbed Fourier transform-infrared (FTIR) spectroscopy of fresh and 850 °C steamed zeolite Cu-ZSM-5 under CO pressure of e) 0.015 mBar, f) 0.1 mBar and g) 0.5 mBar at liquid N_2_ temperature. Blue and red lines represent fresh and 850 °C steamed zeolite Cu-ZSM-5, respectively.

Another important functional moiety in fresh zeolite Cu-ZSM-5 is the isolated, exchanged Cu^2+^ sites located near framework Al for charge compensation. The most direct observation of structural changes upon steaming is the formation of Cu-based nanoparticles as shown in the transmission electron microscopy (TEM) images (Fig. 2). With increasing steaming temperature, the nanoparticles were more evident throughout the whole zeolite particles. In the 850 °C steamed Cu-ZSM-5, nanoparticles with particle sizes of 2–5 nm were prone to migrate and aggregate on the surface of zeolite particle, which is in agreement with a scanning transmission X-ray microscopy (STXM) study that revealed Cu zoning on the edge of individual catalyst particles in steamed zeolites.^15^ The agglomerated Cu species have a less reducible nature than isolated Cu^2+^, as indicated by the higher reduction temperature in the steamed Cu-ZSM-5 observed in H_2_-temperature programmed reduction (H_2_-TPR) (Fig. S6†). Moreover, the broadening of the reduction peaks in the steamed Cu-ZSM-5 also suggests the increasing diversity of Cu species produced by the steaming process.

CO is a universal probe molecule in FTIR spectroscopy experiments to detect the metal sites by charge donation/back-donation between metal center and CO molecule. The interaction of CO with Cu^2+^ is weak, and therefore only Cu^+^ and the hydroxyl group could be probed by CO in Cu-zeolites.^24^Fig. 2e–g shows the FTIR spectra with different CO coverages. One of the differences between fresh and steamed Cu-ZSM-5 in CO-adsorbed FTIR spectra is the stronger peak intensities found in the fresh Cu-ZSM-5, indicating larger numbers of available sites for CO adsorption in the fresh catalyst. The adsorption band of cuprous mono-carbonyls adducts centered at 2159 cm^−1^ coordinated up to three CO molecules with increasing CO pressure, which is well-documented.^25^ This probed Cu^+^ originated from [CuOH]^+^, which experienced auto-reduction during the dehydration pre-treatment under high vacuum.^26^ The loss of [CuOH]^+^ was confirmed in the steamed Cu-ZSM-5 in CO-adsorbed FTIR spectroscopy, and it was accompanied by the co-existence of another cuprous site coordinated with CO with a lower C–O frequency of 2146 cm^−1^, which was also reported in zeolite Cu-ZSM-5 with high Cu-exchanged levels.^27^ This cuprous site had higher coordinative saturation since only the mono-carbonyl was observed. When the CO dosage was high, the CO adsorption on the silanol became detectable only on the steamed catalyst,^28^ consistent with the observation of abundant internal silanol groups in the 850 °C steamed zeolite Cu-ZSM-5.

**Fig. 3:**
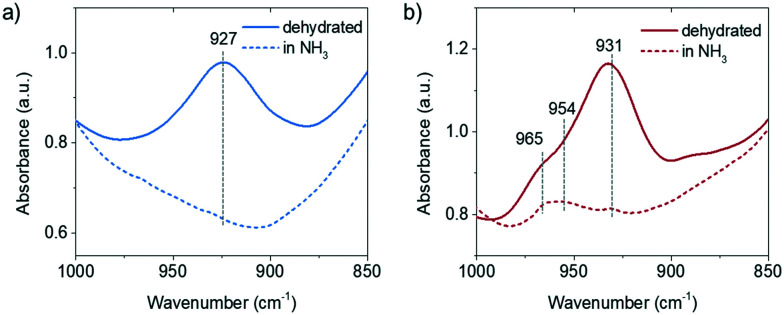
Diffuse reflectance infrared Fourier transform spectroscopy (DRIFTS) data of perturbed framework T–O–T vibration of a) fresh and b) 850 °C steamed Cu-ZSM-5 after dehydration and drying under NH_3_/He flow.

The perturbed framework T–O–T vibration is directly influenced by the interaction between the Cu ion and the framework. Fig. 3 shows the perturbed framework vibration of fresh and 850 °C steamed Cu-ZSM-5 after dehydration and subsequent NH_3_ treatment. The background spectrum was recorded for the hydrated form of zeolites as fully hydrated Cu^2+^ is mobile.^29^ The ammoniated Cu^2+^ hardly interacts with the zeolite framework, showing no perturbed T–O–T band. Upon removal of NH_3_, the Cu^2+^ is stabilized by the framework oxygen and consequently perturbs the framework T–O–T vibration. The perturbance of the framework generally depends on the charge of the interacting cation such that the higher the net charge of the interacting cation, the lower is the value of the T–O–T vibration, because a stronger interaction between opposite charges weakens the original framework vibration to a greater extent.^30^ The transformation from the framework stabilized Cu^+^ to Cu^2+^ causes the band shift of the asymmetric T–O–T vibration from 970 to 910 cm^−1^.^31,32^ The ∼930 cm^−1^ and ∼950 cm^−1^ bands have been assigned to bare Cu^2+^ and [Cu^2+^O^−^]^+^/[Cu^2+^OH^−^]^+^/O_2_-associated Cu^+^, respectively.^30,33^ In the fresh zeolite Cu-ZSM-5, [CuOH]^+^ was not shown in the perturbed framework vibration band, although its existence was clearly indicated by the CO-FTIR results and its OH stretching band at 3660 cm^−1^. The signal from the [CuOH]^+^ perturbance might be covered by the strong and broad band originating from bare Cu^2+^. However, in addition to the Cu^2+^ and Cu^+^ perturbed vibrational modes, the ammoniation process unveiled the 954 cm^−1^ band in the 850 °C steamed Cu-ZSM-5 although it lost the isolated [CuOH]^+^. Only the isolated or clustered Cu ions influence the perturbed framework vibration by ligand removal or addition, because interaction between large particles and zeolite framework could be hardly affected by replacement of ligands. The 954 cm^−1^ band is hereby supposed to be relative to the charged Cu oligomers/clusters [Cu_*x*_(OH)_2*x*−1_]^+^ that could interfere with the framework vibrations. The adjacency of the hydroxyl group to the Cu^2+^ is later implied by *operando* DRIFTS results.

Both NO oxidation and NH_3_ oxidation have been under investigation in Cu-exchanged zeolites suggesting the potential contribution of isolated sites, such as Cu^2+^ and [CuOH]^+^, to the side reactions observed in the catalytic test.^14,21,34^ However, with the steaming-induced local damage of the zeolite framework and the formation of [Cu_*x*_(OH)_2*x*−1_]^+^ oligomers/clusters, the NO conversion above 250 °C dropped due to the large contribution from unselective NH_3_ oxidation. The detrimental effect of Cu_*x*_O_*y*_ clusters/nanoparticles on the NH_3_-SCR reaction has been demonstrated to promote NH_3_ oxidation, and promising NH_3_ conversion was even observed over a physical mixture of CuO and H-SAPO-34.^17,20,35^

### 2.3 Cu^2+^ dynamics unravelled by *operando* UV-vis diffuse reflectance spectroscopy

The Cu aggregation in the steamed Cu-ZSM-5 zeolites was proposed to be responsible for the occurrence of the unselective NH_3_ oxidation reaction that caused the unusual NO and NH_3_ conversion in NH_3_-SCR. However, a more detailed structural correlation of the deactivated component contributions to the loss of NH_3_-SCR activity and the promotion of side reaction NH_3_ oxidation has not yet been well-understood. This requires real-time monitoring of the catalysts in a reaction to establish the structure–activity relationships for further understanding of the key structure involved in the reaction. One of the most facile means to study transition metals under working conditions is UV-vis DRS employing a high temperature UV-vis optical fibre probe.^36^

#### 2.3.1 Replacement of ligands in the Cu complex

**Fig. 4:**
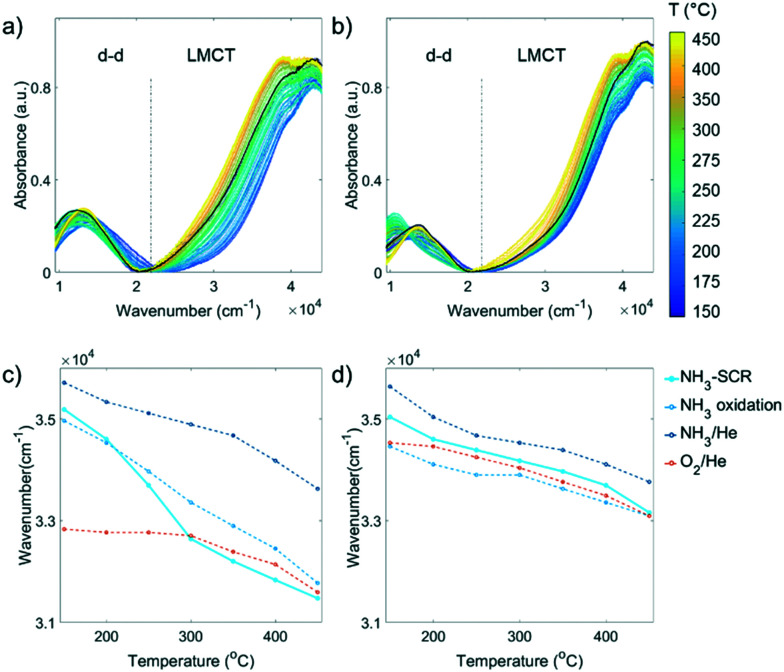
*Operando* UV-vis diffuse reflectance spectroscopy (DRS) data of the a) fresh and b) 850 °C steamed zeolite Cu-ZSM-5 during the NH_3_-selective catalytic reduction (SCR) of NO. The black solid line is the spectrum recorded at 150 °C in O_2_/He before the reactants were fed. The wavenumber at half height of the ligand-to-metal charge transfer (LMCT) band at *ca.* 39 000 cm^−1^ in different reaction conditions was obtained at steady state for c) fresh and d) 850 °C steamed Cu-ZSM-5.

Generally, probing the transition metal Cu with UV-vis DRS gives rise to ligand field induced d–d transitions determined by the number and position of atoms in the first coordination sphere, as well as a ligand–metal charge transfer (LMCT) band influenced by the optical electronegativity between the ligand and Cu.^37^ UV-vis diffuse reflectance spectra were recorded during the NH_3_-SCR reaction on fresh and 850 °C steamed zeolite Cu-ZSM-5, and the results are shown in Fig. 4a and b. The starting spectrum is the dehydrated Cu-ZSM-5 measured before the NH_3_-SCR reaction. A LMCT band with a lower wavenumber in the fresh Cu-ZSM-5 implies Cu^2+^ had an overall stronger interaction with its surrounding O^2−^ compared to the steamed zeolite. Upon reaction, the fresh and steamed zeolite Cu-ZSM-5 follow very similar trends in LMCT transition. The band position had a blue shift once the reactant gases were introduced, and the band position shifted to a lower wavenumber during the entire reaction process. In the NH_3_-SCR reaction and its side reactions, N and O are the only two elements that need to be considered as the atom in the first coordination shell of Cu^2+^. Substitution of O-oriented ligands to N-oriented NH_3_ ligands drives the blue shift of LMCT band because NH_3_ has a smaller optical electronegativity compared to oxygen-oriented ligands including H_2_O, O^2−^, OH^−^ and NO_*x*_^−^, which generates a greater difference in energy level from Cu^2+^.^37–39^ The gradual red shift of the LMCT band with increasing reaction temperature is due to the removal of NH_3_ and the stronger interaction with coordinated O^2−^, *i.e.* the higher degree of covalency in the ligand–metal bond.^25^

To gain an intuitive look into the replacement of ligands in the reaction process, the wavenumber at half height of the LMCT maximum was followed in the NH_3_-SCR reaction, by comparing to that of inflow of O_2_/He, NH_3_/He and NH_3_ oxidation feeds (Fig. 4c and d). In the fresh zeolite Cu-ZSM-5, the reaction could again be clearly divided into two regimes including the low-temperature (150–250 °C) and high temperature (300–450 °C) NH_3_-SCR mechanism according to the position of the LMCT band half height. In the low temperature regime, the position of the LMCT band is shown at a high wavenumber, resembling that of the NH_3_ oxidation condition and being close to the LMCT band position in NH_3_/He, while at a temperature higher than 300 °C, the evolution of the LMCT band position is similar to that found in oxidative gases. This strongly suggests the predominant replacement of the first coordination shell atom to isolated Cu^2+^ from N to O during the reaction, in agreement with similar findings revealed by *in situ* XAS and the proposed different reaction mechanism in low- and high-temperature NH_3_-SCR.^40^ In contrast, a less significant shift of the LMCT band during the reaction was observed in the steamed zeolite Cu-ZSM-5, suggesting a less notable change of the coordinated ligands because fewer Cu^2+^ sites were available to take part in the reaction on the surface of [Cu_*x*_(OH)_2*x*−1_]^+^ oligomers/clusters. No clear demarcation line between the low and high temperature regime was found in the steamed sample, though it had been in the fresh Cu-ZSM-5 zeolite material. The LMCT band positions in NH_3_-SCR reaction and O_2_/He environments are similar, indicating the dominant coordinated ligand was O^2−^ in the steamed Cu-ZSM-5. However, the LMCT band position under NH_3_-SCR reaction conditions lies between its position in the NH_3_/O_2_ environment and NH_3_/He environment, and thereby, the coordination with NH_3_ cannot be ruled out.

#### 2.3.2 Appearance of pseudo-tetrahedral Cu^2+^ in low temperature NH_3_-selective catalytic reduction of NO

**Fig. 5:**
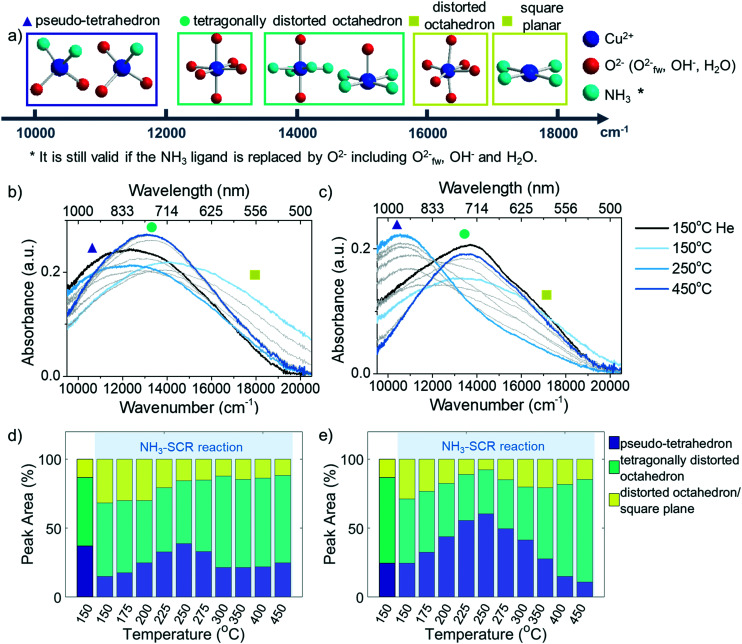
a) Schematic of the possible Cu-complexes in zeolite Cu-ZSM-5 in the NH_3_-SCR reaction and the approximate positions in wavenumbers of their corresponding bands, estimated based on ligand-field imposed d–d splitting of Cu^2+^. O^2−^_fw_ represents framework oxygen. The d–d transition band and the peak fitting results of the (b and d) fresh and (c and e) 850 °C steamed Cu-ZSM-5 zeolites collected at steady-state at each temperature during the NH_3_-SCR reaction performance test. The bands in grey color are collected in intermediate reaction temperatures between 150–450 °C. The positions of both main peak and shoulder are indicated in (a) by a solid triangle (Prussian blue), a circle (cyan) and a square (yellow), respectively.

The d–d transition region from the UV-vis diffuse reflectance spectrum provides information on the geometric structure of Cu^2+^. Incorporation of ligands in different spatial locations has a significant impact on the extent of the splitting of d orbital, which is affected by the extent of interaction between the d orbital and the ligand. Fig. 5b and c show the d–d transition band of fresh and 850 °C steamed zeolite Cu-ZSM-5 at steady-state from 150–450 °C in the NH_3_-SCR reaction. In both data sets, three main absorption bands could be identified in the d–d transition region: below 12 000 cm^−1^, 12 000–16 000 cm^−1^ and above 16 000 cm^−1^. Despite the band broadening and overlapping resulting from the variety of ligand combinations and heterogeneity of Cu^2+^ symmetry, the appearance of two spectroscopic signatures at 10 350 cm^−1^ and 13 700 cm^−1^ are clearly identified in the UV-vis spectra of 850 °C steamed Cu-ZSM-5. The symmetry change of Cu^2+^ was followed using ligand-field theory as well as previous experimental/theoretical studies on the Cu^2+^ UV-vis spectrum.

As indicated by Fig. 5b and c, low, medium, and high wavenumber bands could be identified in the d–d transition region. The adsorption band at ∼12 500 cm^−1^ is the typical band that arises from isolated Cu^2+^ in an octahedral crystal field generated by oxide ligands.^41^ The [Cu(H_2_O)_6_]^2+^ is the tetragonally distorted complex in the fully hydrated zeolite due to framework confinement. The dehydration process generates the axial elongation of Cu^2+^ tetragonal bipyramidal geometry upon water removal and framework attachment, which causes further splitting of octahedral ligand field and therefore the slight blue shift of the band maximum of the d–d transition.^25^ The Cu^2+^ complex in a square-planar environment has a larger splitting of the d orbital compared to octahedral, according to crystal-field theory. The simulated d–d transition band energy for the near square planar complex [Cu(NH_3_)_4_]^2+^ with optimized structure presented its maximum absorption at 19 800 cm^−1^, which was in agreement with experimental observation.^42,43^ The high wavenumber shoulder appeared in the low temperature NH_3_-SCR reaction shown in Fig. 5b and c centered at *ca.* 17 000 cm^−1^, suggesting the likely incorporation of a weaker field ligand O^2−^ that might cause the redshift of the peak, *i.e.* the formation of [Cu(NH_3_)_4_(H_2_O)_*x*_]^2+^ (*x* = 1,2). The high wavenumber band could also stem from the Cu^2+^ complex with low symmetry that enlarges the splitting of the d orbital. At a reaction temperature between 175–300 °C, the 10 350 cm^−1^ band became apparent especially in the steamed zeolite Cu-ZSM-5. The lower wavenumber feature of such a band implicated the smaller splitting of d orbital compared to octahedral symmetry, probably due to the tetrahedral crystal field environment (Δ_T_ ≈ 4/9 Δ_O_).^44^ The 10 350 cm^−1^ band was related to the coordinated NH_3_ since it was also present in NH_3_/O_2_ and NH_3_/He environments (Fig. S9†).

Partial de-ammoniation of Cu^2+^ caused a transition to square planar geometry, an extreme case of tetragonal distortion from octahedral symmetry, which is high in energy and is prone to relax to tetrahedral symmetry. In fact, the pseudo-tetrahedral Cu^2+^-complex could be expected in zeolite Cu-ZSM-5. The Cu^2+^ lying in a defect site or on the surface of [Cu_*x*_(OH)_2*x*−1_]^+^ oligomers/clusters probably had then required steric hinderance for the coordinating ligand to reach octahedral symmetry; instead, the more stable tetrahedron is favoured. Indeed, the contribution of this low-wavenumber band increases with the steaming severity (Fig. S10†). A similar band maximum at 11 000 cm^−1^ has been reported in ammoniated Cu-exchanged zeolite Y with a low coordination number.^45^ This low-frequency band has been hypothesized as the O_3_–Cu^2+^–NH_3_ structure in de-ammoniated Cu-zeolite, which was proven by multiconfigurational perturbation theory based simulation and an electron paramagnetic resonance (EPR) study.^42,46^ Hence, the low frequency feature is also proposed to be the pseudo-tetrahedral Cu^2+^ with a mix of NH_3_ and O^2−^-oriented ligands (O_3_–Cu^2+^–NH_3_ or O_2_–Cu^2+^–(NH_3_)_2_).

To follow the evolution of Cu-complexes throughout the NH_3_-SCR reaction, the d–d transition bands were fitted with three Gaussian functions by restricting fitting model parameters such as their wavenumber position based on the inspection of the eigen spectra from principal component analysis (PCA, Fig. S11†). The such established fitting model was then applied to the entire dataset, and the fitting results can be found in Fig. S12–S14.† The evolution of pseudo-tetrahedral Cu^2+^, tetragonally distorted Cu^2+^ and low symmetrical Cu^2+^ in fresh and 850 °C steamed zeolite Cu-ZSM-5 at steady-state during the NH_3_-SCR reaction are given in Fig. 5d and e. Both Cu-ZSM-5 samples behaved in a similar manner during the reaction when following the peak contributions of pseudo-tetrahedral and distorted octahedron/square planar Cu^2+^ during the reaction. Upon the exposure to the reactant gases, ammoniated Cu^2+^ developed along with the coordination with H_2_O. This feature gradually diminished with increasing reaction temperature (150–250 °C) because of the detachment of NH_3_ from Cu^2+^. At the same time, the contribution of a pseudo-tetrahedral Cu^2+^-complex with mixed NH_3_ and O^2−^-oriented ligands increased sharply. When the NH_3_-SCR reaction took place above 250 °C, the amount of pseudo-tetrahedral Cu^2+^-complex with mixed ligands started to decrease due to the continuous freeing of coordinated NH_3_. In this scenario, the d–d transition band of UV-vis diffuse reflectance spectra in the NH_3_-SCR reaction feed were eventually identical to that under O_2_/He flow (Fig. S15†), suggesting complete removal of coordinated NH_3_ and the presence of tetragonally distorted octahedral Cu^2+^ with an O^2−^ ligand.

It is important to note that the change of Cu^2+^ geometry was due to the reaction-related dynamic but not the irreversible change of Cu^2+^ structure since the NO/NH_3_ conversion as well as the geometry were unchanged when the NH_3_-SCR reaction was conducted in a cyclic manner (Fig. S16†). In the low temperature regime (<250 °C), the NH_3_-SCR reaction was the preferential reaction according to the catalytic results. The mobile [Cu(NH_3_)_4_]^2+^ complex is the proposed catalytic active site that is ready to react with NO at a reaction temperature under 250 °C.^40,47,48^ A higher portion of NH_3_-solvated Cu^2+^ observed in the fresh zeolite Cu-ZSM-5 was attributed to the higher NO conversion at a low reaction temperature compared to the steamed Cu-ZSM-5. At a reaction temperature of 150–250 °C, the adsorbed NH_3_ either desorbed or reacted with intermediates, resulting in partially de-ammoniated Cu^2+^ with pseudo-tetrahedral symmetry, which was simultaneously coordinated with O^2−^ or with the intermediate NO_*x*_^−^.^49,50^ The accumulation of Cu^2+^ in pseudo-tetrahedral symmetry with coordinated NH_3_, which is stable below 250 °C, limits the NO and NH_3_ conversion to a great extent particularly in steamed Cu-ZSM-5. As the reaction temperature increased from 250 °C, the coordinated NH_3_ in pseudo-tetrahedral Cu^2+^ started to disassociate, which could be proven by the desorption of NH_3_ adsorbed on Cu^2+^ with Lewis acidity (Fig. S7†). However, such desorption of NH_3_ from pseudo-tetrahedral Cu^2+^ provoked unselective NH_3_ oxidation rather than the NH_3_-SCR reaction, which is clear in the steamed Cu-ZSM-5 from the rapid increase of NH_3_ conversion and a dramatic drop of NO conversion between 250–300 °C. Finally, in high-temperature NH_3_-SCR above 300 °C, the adsorption of NH_3_ weakens, rather, the fully de-ammoniated Cu^2+^ tends to anchor on the framework O^2−^ with coordination of four,^42,51^ resulting in the identical geometric structure as it has in O_2_/He flow. The Cu^2+^ complex with an O^2−^-directing ligand is the key species for the high temperature NH_3_-SCR reaction in fresh and steamed Cu-ZSM-5, allowing the maximum Cu^2+^ coordination number to be a distorted octahedron by interaction with external ligands, for example the possible reaction intermediates NO_2_ or NO_*x*_^−^.

Particularly for 850 °C steamed Cu-ZSM-5, the Cu^2+^ geometry is identical in the NH_3_-SCR reaction and unselective NH_3_ oxidation reactions (Fig. S9†), which is strong proof of the great impact of unselective NH_3_ oxidation in the NH_3_-SCR reaction. It also points out the similarity of the Cu^2+^ local structure that is responsible for NH_3_-SCR and unselective NH_3_ oxidation. In the intermediate reaction temperature of 250–300 °C, desorption of NH_3_ mainly took place on the surface of [Cu_*x*_(OH)_2*x*−1_]^+^ oligomers/clusters and was followed by the rapid oxidation into NO, resulting in the sudden increase of NH_3_ conversion.

### 2.4 Adsorption competition revealed by *operando* diffuse reflectance infrared Fourier transform spectroscopy

**Fig. 6:**
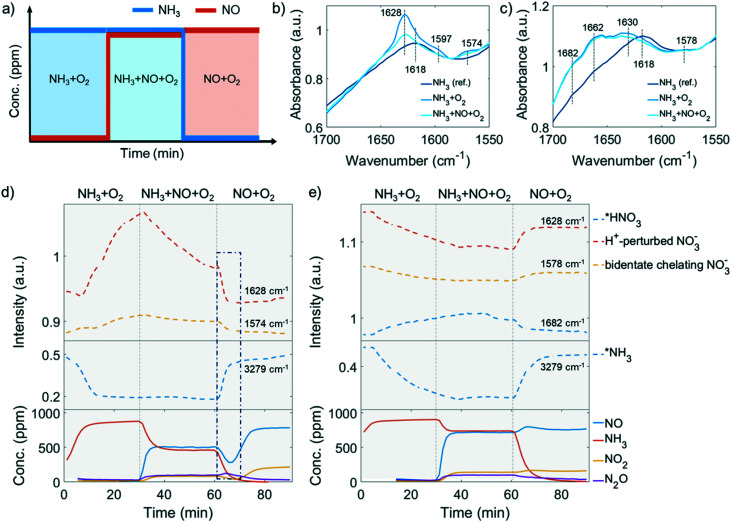
a) Procedure of the *operando* diffuse reflectance Fourier transform spectroscopy (DRIFTS) experiment performed. The experiment was conducted after O_2_ calcination at 550 °C followed by NH_3_ + O_2_, NH_3_ + NO + O_2_ and NO + O_2_ reaction at 250 °C with 1000 ppm of NH_3_ and/or 1000 ppm NO balanced by 5% O_2_/He. The obtained *operando* DRIFTS spectra of surface nitrates in b) fresh and c) 850 °C steamed zeolite ZSM-5. The evolution of selective bands representing surface nitrates (upper panel) and adsorbed NH_3_ (middle panel) were followed and the recorded concentration of effluent gas composition (bottom panel) in the d) fresh and e) 850 °C steamed zeolite Cu-ZSM-5. Missing datapoints at the beginning of the experiment (Fig. 6d and e, bottom panel) are due to values below the limit of detection.

The low-temperature standard NH_3_-SCR reaction (<250 °C) attracts extra attention because improved NO conversion is needed in this temperature range. The NH_3_-SCR catalysts inevitably deactivate from steam produced by fuel combustion. As indicated in the catalytic performance results, 250 °C is a critical point, from which the low-temperature NH_3_-SCR starts to transition to the high-temperature reaction. At this temperature, no apparent side reaction takes place in the NH_3_-SCR reaction even in the 850 °C steamed Cu-ZSM-5, as NH_3_ and NO have the same conversion (Fig. 1d), which is beneficial for investigating the behaviour in NH_3_-SCR without interference. Adsorbed surface species are potentially useful to gain insight into the reaction and deactivation pathways. The experimental protocol for the DRIFTS experiment is described in Fig. 6a. The experiment was conducted at 250 °C after a calcination step. The reaction started with NH_3_ oxidation, followed by NO addition and subsequent NH_3_ removal to achieve NH_3_-SCR and NO oxidation, respectively.

#### 2.4.1 Observed surface species

Fig. 6b and c shows the DRIFTS data recorded on fresh and 850 °C steamed Cu-ZSM-5 after exposure to NH_3_, NH_3_ + O_2_ or NH_3_ + NO + O_2_ flow for 30 min at 250 °C. Surface nitrates produced in the *operando* DRIFTS experiment attract the most attention as they have been proposed as an important reaction intermediate. The vibration originating from adsorbed NO_3_^−^ species on zeolite Cu-ZSM-5 is in the range of 1570–1700 cm^−1^, and the bridging nitrate is found at 1618 cm^−1^ while the bidentate chelating nitrate is identified by 1597, 1578/1574 cm^−1^ bands in former FTIR spectroscopy studies under NO_2_ or NO + O_2_ atmosphere.^11,52–54^ The formation of a bidentate nitrate during NH_3_-SCR has been validated in a density functional theory (DFT) study.^50^ However, it should be noted that Lewis acid-adsorbed ammonia (L-NH_3_) has its N–H bending frequency at ∼1620 cm^−1^, therefore care should be taken to the assignment of the 1618 cm^−1^ band. The assignment of the 1628 cm^−1^ band is less conclusive. It is often attributed to the *ν*_N=O_ of a bridging nitrate where two vicinal Cu atoms are required to anchor two nitrate O^2−^.^55^ With this postulation, a stronger 1628 cm^−1^ band should be found in the steamed zeolite Cu-ZSM-5 as more adjacent Cu atoms are available when Cu aggregation has happened. However, this is contradicted by the fact that the 1628 cm^−1^ band is a shoulder in the steamed Cu-ZSM-5, while it is an intense peak in its fresh counterpart (Fig. 6b and c). Alternatively, the 1628 cm^−1^ feature has been proposed to be the proton-perturbed chelating nitrate whose N=O vibration is affected by the nearby Brønsted acid site; this assignment was shown by a systematic FTIR spectroscopy study of surface Cu^2+^(N,O) species on Cu-CHA,^56^ which could explain why the pronounced 1628 cm^−1^ band was more intense on the fresh Cu-ZSM-5 with well-defined Brønsted acid sites. The 1662 cm^−1^ and 1682 cm^−1^ band related to the adsorbed NO_2_ are unique in the 850 °C steamed zeolite Cu-ZSM-5, where the former one can be attributed to adsorbed NO_2_ while the latter one is from the protonated NO_2_.^57,58^ It is not surprising to observe NO_*x*_ adsorption on the surface of steamed Cu-ZSM-5 zeolites with abundant hydroxyl groups, which leads to the formation of HNO_*x*_ during the *operando* DRIFTS experiment where NH_3_-SCR, NH_3_ oxidation, or NO oxidation took place. The conversion between nitrous acid and nitric acid was kept in balance depending on the local concentration of NO and NO_2_. Finally, the 1682 cm^−1^ band was tentatively attributed to the adsorbed HNO_3_, considering that HNO_2_ is easily oxidized to HNO_3_ at reaction conditions.

#### 2.4.2 Competitive adsorption of surface species

Ammonia with a lone pair of electrons on the N side is one of the main surface species observed during the *operando* DRIFTS experiment. The typical symmetric and asymmetric stretching modes of adsorbed NH_3_ are in the region of 3400–3000 cm^−1^ with strong absorbance. The band at ∼3180 cm^−1^ together with a 1617 cm^−1^ band are characteristic of L-NH_3_,^53^ which could be observed in fresh and steamed Cu-ZSM-5 zeolites once the catalysts were exposed to NH_3_/O_2_ (Fig. S17 and S18†). The evolution of important DRIFTS bands including N–H stretching of L-NH_3_, perturbed framework T–O–T vibration and surface nitrates were followed and are shown in Fig. S19 and S20.† Particularly, the development of adsorbed HNO_3_, H^+^-perturbed nitrate, chelating bidentate nitrate, as well as adsorbed NH_3_ were selected to show in Fig. 6d and e, together with the real-time concentrations of reactants and products.

**Scheme 1:**
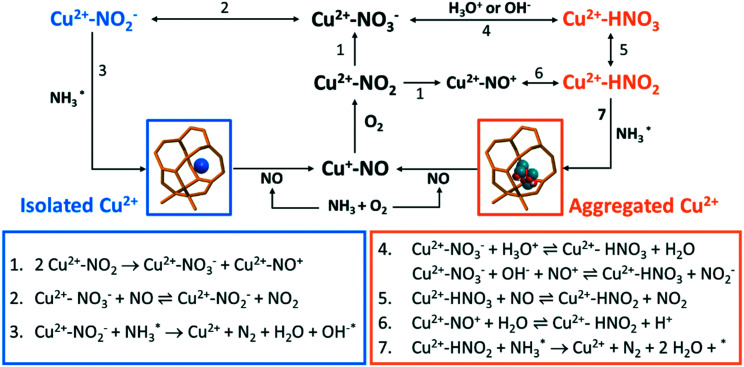
Possible reactions that take place in the low-temperature NH_3_-selective catalytic reduction (SCR) reaction over Cu–zeolites. The blue colour indicates dominant reactions that can take place on isolated Cu^2+^, while the orange colour stands for possible reactions over aggregated Cu^2+^ species, such as [Cu_*x*_(OH)_2*x*−1_]^+^ oligomers/clusters.

In the first step of the *operando* DRIFTS experiment on the fresh Cu-ZSM-5 zeolite, nitrates developed in NH_3_/O_2_ flow (Fig. 6d), suggesting the full oxidation of surface NH_3_, which was also observed in *in situ* FTIR studies on the NH_3_ oxidation reaction.^59,60^ The formed nitrates replaced the pre-adsorbed NH_3_ on Cu^2+^ and weakened the adsorbed NH_3_ signal (Fig. 6 and S17†). The re-appearance of the Brønsted acid site (Fig. S17a†) might be due to the reaction between the Brønsted acid adsorbed NH_3_ (B-NH_3_) and L-NO_3_^−^ followed by restoration of the proton from H-cleavage of NH_3_.^61^ The NH_3_ oxidation reaction was followed by the NH_3_-SCR reaction where NO participated and reacted with surface nitrates, resulting in the formation of NO_2_ and NO_2_^−^ (reaction 2, Scheme 1) that avoid the surface blockage by nitrates.^3^ Unfortunately, we cannot confirm the formation of surface nitrites as the O–N–O stretching frequency was covered by the signal from symmetric stretching of N–H as well as the intense signal from the zeolite structure.^62,63^ In the last step of NH_3_ removal, consumption of surface nitrate species accelerated. The involvement of NO in nitrate depletion is now strongly supported by the simultaneous drop of NO concentration as surface nitrates are decreasing, which is indicated by the blue rectangle in Fig. 6d. Interestingly, once the nitrates were depleted, the Cu^2+^ site is re-occupied by the residual NH_3_ (Fig. 6d, bottom panel). In fact, a similar phenomenon of NH_3_-nitrates competitive adsorption has been reported in Cu-exchanged zeolites, where adsorbed nitrates and NH_3_ on Lewis acid sites could be replaced by each other depending on reaction conditions.^10,64,65^ The Lewis acid, which is isolated Cu^2+^ in our case of fresh Cu-ZSM-5, is thus the suggested main site for NH_3_ adsorption and nitrate formation/adsorption according to the changes of the perturbed framework vibration with the surface species involved (Fig. S17†).

The causes of deactivation were revealed from the *operando* DRIFTS data collected on the 850 °C steamed Cu-ZSM-5 zeolite (Fig. 6e). In NH_3_/O_2_ flow, adsorbed NH_3_ and nitrates developed in the initial stage, followed by the disappearance of both surface species along with the increased adsorbed nitric acid (Fig. 6e, S18a and c†). No significant change of surface species was observed upon the subsequent addition of NO in the second step. Similarly, the NH_3_ desorption in NH_3_/O_2_ flow could be explained by the competitive adsorption between NH_3_ and nitric acid, because these two species exhibited opposite trends throughout the whole DRIFTS experiment (Fig. 6e). The production of nitric acid is related to the attenuation of surface nitrates (Fig. 6e), which can react with the adjacent proton H^+^/hydronium or surface hydroxyl group to from nitric acid (reaction 4, Scheme 1).^53^ The observed adsorbed NO_2_ was the precursor for nitric acid formation, showing good agreement with an *in situ* FTIR study conducted on hydroxyl-rich or hydrated silica,γ-Al_2_O_3_ and TiO_2_.^58,66,67^ In the final step in NO/O_2_, analogous to the nitrate depletion in the fresh sample, nitric acid was reduced by NO (Fig. 6e), producing NO_2_ that could turn into nitrate by disproportionation (reaction 5 and 1, Scheme 1).^58,68^ The surface coverage of nitrates and nitric acid results from the competition between their formation and consumption.

The competitive adsorption of NH_3_ and surface nitrates/nitric acid occurred on both fresh and 850 °C steamed Cu-ZSM-5 zeolites. With the replacement of adsorbed NH_3_, nitrates/nitric acid was generated with the appearance of perturbed framework vibration by Cu^2+^ (in the fresh Cu-ZSM-5) and [Cu_*x*_(OH)_2*x*−1_]^+^ oligomers/clusters (in the 850 °C steamed Cu-ZSM-5) as demonstrated in Fig. S17 and S18.† Not all the [CuOH]^+^ sites were involved in the reaction since they were partially preserved in NH_3_-rich flow and were not perturbed by surface nitrates (Fig. S17a, d and g†). Nitric acid rather than nitrates was the more stable intermediate that was more ready to react with adsorbed NH_3_ in the 850 °C steamed Cu-ZSM-5.

#### 2.4.3 Nitrate mediated reaction network

The main reaction discussed here is the standard NH_3_-SCR reaction, which has competition from side reactions that bring about an intricate reaction system. Adsorbed neutral and ionic NO_*x*_ were the most common spectator species in the *operando* DRIFTS experiment, and the plausible interconversion between adsorbates is summarized in Scheme 1.^3,53,68,69^ It is noted that all reactions in Scheme 1 can happen in fresh and steamed Cu-ZSM-5 zeolites, but reaction routes involving HNO_*x*_ are more privileged in the steamed Cu-ZSM-5 due to the presence of more aggregated Cu sites.

The coupling of NO oxidation and NH_3_ oxidation with the standard NH_3_-SCR reaction is through surface nitrates, which are formed from adsorbed NO_2_. NO_2_ can be formed through several reaction pathways at NH_3_-SCR reaction conditions according to the catalytic results (Fig. 1). NO_2_ is one of the byproducts generated from the participation of either NO oxidation reaction below 250 °C or unselective NH_3_ oxidation reaction above 250 °C. The NO oxidation reaction produces NO_2_, which is formed *via* reaction between dissociated O_2_ and loosely adsorbed NO according to a detailed kinetic model of NO oxidation.^70^ For NH_3_ oxidation, although its reaction mechanism is still under debate, a two-step reaction pathway named ‘the internal SCR mechanism’ has been proposed where NH_3_ is first oxidized to NO_*x*_ followed by the NH_3_-SCR reaction.^1,61^

In the NH_3_-SCR reaction, the NO oxidation with molecule O_2_ into nitrates through the formation of NO_2_ has been stressed as it is suggested as a rate-determined step.^50^ The formation of NO_2_ promotes the formation of surface nitrates and meanwhile boosts the reoxidation of Cu^+^ to Cu^2+^ in the oxidation half cycle.^71–74^ A Cu monomer in Cu-exchanged zeolites has been reported as the NO_2_ adsorption site, enabling NH_3_-SCR reaction to proceed with NO_2_ intermediate.^75^ Nevertheless, NO_2_ could be also detected as an undesired side product. Upon encountering the hydroxylated or hydrated surface, nitrates could be protonated to form surface nitric acid, which happened in the 850 °C steamed Cu-ZSM-5 as shown in Fig. 6c. However, the surface nitrite rather than nitrate is the key intermediate for the desired N_2_ production, demonstrating the importance of nitrate–nitrite equilibrium (reaction 2 and 4, Scheme 1) which greatly influences the reaction selectivity. Shifting the equilibrium towards nitrite formation was witnessed in both fresh and steamed Cu-ZSM-5 zeolites, deduced by the nitrate depletion with the introduction of NO in NH_3_/O_2_ feed in the *operando* DRIFTS experiments. A similar founding was also described in a combined FTIR-XAS study on zeolite Cu-SSZ-13.^76^ Successive reaction of nitrites/nitrous acid with L-NH_3_ lead to products formation (reaction 3 and 7, Scheme 1). Participation of B-NH_3_ was not observed based on the *operando* DRIFTS data because of the observation of well-preserved of Brønsted acid sites, although some previous studies suggested the surface NH_4_NO_3_/NH_4_NO_2_ is reaction intermediate that decompose to N_2_O/N_2_ at reaction temperatures.^53,72,77^

#### 2.4.4 Important mechanistic implications

Several mechanistic implications related to the low temperature NH_3_-SCR reaction could be obtained from the *operando* DRIFTS data. Firstly, adsorbed NH_3_ reacts with NO_*x*_*via* a Langmuir–Hinshelwood mechanism that surface reaction takes place between two adsorbed species.^1^ It is concluded based on the fact that the adsorbed NH_3_ did not directly react with gaseous NO/O_2_ indicated in the third step in the DRIFTS experiment, but react with surface nitrates, as inferred from the restoration of the Brønsted acid site and [CuOH]^+^ after the NO_3_^−^ developed in the NH_3_/O_2_ step (Fig. 6d and S17†). Secondly, the participation of NO in the NH_3_-SCR reaction can happen *via* the reaction with surface nitrates as explained by the nitrate depletion after NO addition to the NH_3_/O_2_ feed in *operando* DRIFTS experiment (Fig. 6d). Surface nitrates are more likely to form from NO/O_2_ in the steamed Cu-ZSM-5 with [Cu_*x*_(OH)_2*x*−1_]^+^ oligomers/clusters proven by the observation of nitrate development in NO/O_2_ feed (Fig. S18i†). Finally, no direct participation of a Brønsted acid was found under NH_3_-SCR reaction conditions at steady state, since the OH stretching signal of Brønsted acid sites was preserved (Fig. S17d†). However, the proton from Al–O(H)–Si perturbed the surface nitrates, weakened the OH stretching and caused the redshift of its original vibration of 3610 cm^−1^ after calcination (Fig. S5†) to 3602 cm^−1^ when surface nitrates were formed (Fig. S17†). In this way, with the perturbance from the Brønsted acid, the structure of surface nitrates resembles nitric acid, which probably facilitates the reaction with NH_3_.

### 2.5 Structure–intermediate–performance relationship

A structure–intermediate–performance relationship can be established to elucidate the reasons behind the high activity at low temperature for the NH_3_-SCR reaction in fresh Cu-ZSM-5 and the undesirable side reactions in the steamed Cu-ZSM-5 by correlating the results from the *operando* UV-vis DRS and DRIFTS experiments. NH_3_ did not completely desorb from Lewis sites according to NH_3_-TPD (Fig. S7†) and *operando* UV-vis diffuse reflectance spectra at a low reaction temperature (Fig. 5).

Adsorbed NH_3_ is prerequisite for the low temperature reaction (<250 °C), which is ready to react with the surface nitrites/nitrates intermediates. The higher coordination number of Cu^2+^ in tetragonally-distorted octahedral symmetry in the fresh Cu-ZSM-5 ensures that enough empty orbitals are available for stabilizing nitrites/nitrates, together with NH_3_ ligands judged by the evolution of the LMCT band. It is noted that the complete desorption of L-NH_3_ happened at ∼400 °C in NH_3_-TPD. Therefore, low surface coverage of L-NH_3_ at 250 °C during the NH_3_-SCR reaction observed in *operando* DRIFTS experiment (Fig. S17d†) implied the highly active nature of adsorbed NH_3_ on isolated Cu^2+^ for the formation of nitrites/nitrates intermediates.

As for the 850 °C steamed Cu-ZSM-5 zeolite, NO and NH_3_ conversion was low at 150–250 °C during which the pseudo-tetrahedral Cu^2+^ (O_3_–Cu^2+^–NH_3_ or O_2_–Cu^2+^–(NH_3_)_2_) accumulated likely due to the nitrates/nitric acid adsorbed [Cu_*x*_(OH)_2*x*−1_]^+^ oligomers/clusters coordinated with a NH_3_ ligand. A slower reaction rate between adsorbed NH_3_ and nitrates/nitric acid was found on [Cu_*x*_(OH)_2*x*−1_]^+^ oligomers/clusters because of the co-existence of these surface species. And the surface coverage of nitrites/nitrates intermediates was also lower in the steamed Cu-ZSM-5 zeolite compared to that of in its fresh counterpart (Fig. 6d and e). When the L-NH_3_ desorption started from 250 °C, the Cu^2+^ in pseudo-tetrahedral symmetry also began to disappear due to NH_3_ removal (Fig. 5c and e). In the steamed Cu-ZSM-5, the freed NH_3_ was expeditiously oxidized and released NO from the surface of [Cu_*x*_(OH)_2*x*−1_]^+^ oligomers/clusters because of its weaker coordinating ability to stabilize reaction intermediates. This caused the peaked conversion of NH_3_ and the undesired NO production at 250–300 °C. Additionally, in the 850 °C steamed Cu-ZSM-5, the same active unit and the same intermediate resulted in the same pseudo-tetrahedral Cu^2+^ structure with mixed NH_3_ and nitrates/nitric acid in the NH_3_-SCR and NH_3_ oxidation reaction process. The [Cu_*x*_(OH)_2*x*−1_]^+^ oligomers/clusters could be further aggregated into Cu(OH)_2_, which was recently proposed as the precursor of the inactive CuAl_2_O_4_ species.^9^

### 2.6 Practical implication of detected acidic products

Understanding the relationship between the NH_3_-SCR reaction and its side reactions including NH_3_ oxidation and NO oxidation is required to address the practical problems that the NH_3_-SCR catalysts, which are Cu-based zeolites, inevitably experience during hydrothermal aging in the exhaust pipe of a vehicle. This causes irreversible structural damage that starts from local degradation of framework Al or Cu migration/aggregation. Changes in the structural properties tune the reaction direction and consequently the reaction activity and selectivity. Protonated NO_2_ was identified as a significant spectator based on the *operando* DRIFTS experiment over the steamed zeolite Cu-ZSM-5, suggesting a hydroxylated environment around the NO_2_ adsorption site, for example the [Cu_*x*_(OH)_2*x*−1_]^+^ oligomers/clusters found in this study. Moreover, the effects of abundant internal silanol groups generated from the steaming process cannot be ruled out, which is facilitated for H_2_O adsorption *via* hydrogen bonding. Hydroxyl groups and H_2_O should be considered in the nitrate-mediated reaction network, resulting in the formation of surface HNO_3_ and HNO_2_ according to reactions 4–6 in Scheme 1. The acid–base reaction between HNO_*x*_ and NH_3_ occurs to form NH_4_NO_*x*_ which is capable of decomposing into N_2_ or N_2_O.^53,78,79^ It should be stressed that surface HNO_3_/HNO_2_ is not exclusive to the steamed zeolite since it has also been reported to be involved in elementary steps in the NH_3_-SCR reaction in a microkinetic model over Cu-ZSM-5.^78^

Although NO_2_ incorporates in the reaction through fast NH_3_-SCR or is converted into surface NO_*x*_^−^ or HNO_*x*_, excessive NO_2_ was still detected from the outlet even from the fresh zeolite Cu-ZSM-5. Considering the practical reaction conditions after a vehicle engine, the limited amount of acidic NO_2_ byproduct can convert to nitric acid in H_2_O vapor (4NO_2_ + 2H_2_O + O_2_ ⇌ 4HNO_3_) produced by diesel combustion, while the nitric acid can also be reversely decomposed to NO_2_ at relatively high operational temperatures. Therefore, another adsorbent/catalyst to trap or further remove of possible undesired acidic components is still necessary after the NH_3_-SCR unit in an automotive emission control system. Alkali or alkaline earth metal oxide based catalysts are promising lean NO_*x*_ trap (LNT) catalysts,^80^ which can be placed at the exist of the NH_3_-SCR unit to limit the emission of acidic byproducts.

## Conclusions

Side reactions in the NH_3_-SCR reaction, such as NO oxidation and NH_3_ oxidation, should not be neglected, especially when considering the practical application of the Cu–zeolite-based catalysts in the tailpipe exhaust treatment of diesel vehicles. The contribution of NO oxidation was found at low reaction temperatures (<250 °C), which is considered an essential temperature range for Cu^+^ reoxidation in the redox cycle. When the reaction temperature is higher than 250 °C, the contribution from unselective NH_3_ oxidation accelerated and produced NO, resulting in a ‘dip’ shape of the NO conversion curve in the NH_3_-SCR reaction in steamed Cu-ZSM-5 zeolites. The occurrence of unselective NH_3_ oxidation was found to be more significant in the more severely steamed Cu-ZSM-5 samples, which was ascribed to the aggregation of Cu whose structure is postulated to be [Cu_*x*_(OH)_2*x*−1_]^+^ oligomers/clusters.

Combining the results from the *operando* UV-vis DRS and DRIFTS experiments, we propose that Cu^2+^ probably degraded into [Cu_*x*_(OH)_2*x*−1_]^+^ oligomers/clusters, which can further grow into Cu(OH)_2_ nanoparticles in the steamed zeolite Cu-ZSM-5. The dynamic changes in the symmetry of the Cu^2+^ complex revealed *via operando* UV-vis DRS show the structural reason for the high NH_3_-SCR reaction activity of the fresh Cu-ZSM-5 and the deactivation of steamed Cu-ZSM-5. Octahedral Cu^2+^ with a coordination number of six can be formed during the NH_3_-SCR reaction in the fresh Cu-ZSM-5, facilitating the reaction with a high surface coverage of intermediates. The low temperature reaction showed the preference for NH_3_ coordination, which is replaced by O^2−^-oriented ligand coordination at elevated reaction temperature, confirming different reaction mechanisms in low- and high-temperature NH_3_-SCR. There is a more pronounced formation of pseudo-tetrahedral Cu^2+^ in the steamed Cu-ZSM-5 during the low temperature reaction. The pseudo-tetrahedral symmetry is closely related to partially de-ammoniated Cu^2+^ and its adsorption on the surface of [Cu_*x*_(OH)_2*x*−1_]^+^ oligomers/clusters. The relaxation of this distorted structure by further removal of NH_3_ ligand brings about the undesired NH_3_ oxidation reaction. It should be noted that the same geometric structure of a Cu^2+^ center is shared with low-temperature NH_3_-SCR and NH_3_ oxidation, but higher NH_3_ conversion was found in the NH_3_-SCR reaction, stressing the important role of NO in NH_3_-SCR reaction.

The performed *operando* DRIFTS experiments suggest that isolated Cu^2+^ in the fresh Cu-ZSM-5 and the [Cu_*x*_(OH)_2*x*−1_]^+^ oligomers/clusters in the steamed Cu-ZSM-5 are the main sites participating in the NH_3_-SCR reaction up to 250 °C. This can be concluded from the competitive adsorption between NH_3_ and surface nitrates/nitric acid at 250 °C, because they share the same adsorption sites on Lewis acid sites. Surface nitrates are the key surface species to bridge the NH_3_-SCR, NH_3_ oxidation and NO oxidation reactions. However, surface nitric acid was more prevalent in the steamed Cu-ZSM-5 because of the presence of high density of hydroxyl groups. The high surface coverage of nitrates/nitric acid was reconciled by the reaction with NO to avoid the surface blockage; this reaction governs the nitrate–nitrite equilibrium that determines the selectivity of the reaction. Additionally, no direct involvement of Brønsted acid sites in the NH_3_-SCR reaction was observed at steady state, instead, the surface nitrates were perturbed by the nearby proton, probably from the Brønsted acid sites. Finally, a structure–intermediate–performance relationship could be established to elucidate the low NH_3_-SCR activity and the ‘dip’ shape of NO conversion curve in the steamed Cu-ZSM-5: the pseudo-tetrahedral Cu^2+^ complex of [Cu_*x*_(OH)_2*x*−1_]^+^ oligomers/clusters with associated NH_3_ and nitrates/nitric acid exhibited low activity below 250 °C due to the relatively strong adsorption of surface species; the further increase in temperature (above 250 °C) released the NH_3_ and directed the unselective NH_3_ oxidation. For practical implications, the formation of adsorbed NO_2_ and surface nitric acid should be considered for a better design of vehicle exhaust control systems to meet the requirement of future stringent regulations.

## Experimental

See ESI.†

## Author contributions

X. Ye designed and performed the experiments, as well as processed the acquired data, and drafted the manuscript. R. Oord and M. Monai participated in the discussion of the results, while J. Schmidt provided scientific suggestions and revised the manuscript. T. Chen, F. Meirer and B. M. Weckhuysen supervised the research and the preparation and writing of the article.

## Conflicts of interest

The authors declare no conflicts of interest.

## Supplementary Material

CY-012-D1CY02348A-s001
