# Supplementary material for: New insights into the NH3-selective catalytic reduction of NO over Cu-ZSM-5 as revealed by operando spectroscopy
Source: Catal Sci Technol. 2022 Feb 28;12(8):2589–603. doi: 10.1039/d1cy02348a (PMC9016411; doi:10.1039/d1cy02348a)
Supplement: CY-012-D1CY02348A-s001 [file CY-012-D1CY02348A-s001.pdf]

Electronic Supplementary Material (ESI) for Catalysis Science & Technology.
This journal is © The Royal Society of Chemistry 2022

Supplementary Information

New Insights into the NH_3-Selective Catalytic Reduction of NO over Cu-ZSM-5 as Revealed by *Operando* Spectroscopy

Xinwei Ye,[a, b] Ramon Oord,[b] Matteo Monai,[b] Joel E. Schmidt,[b] Tiehong Chen,[a] Florian Meirer,[b] and Bert M. Weckhuysen*,[b]

[a.] Institute of New Catalytic Materials Science, School of Materials Science and Engineering, Key Laboratory of Advanced Energy Materials Chemistry (MOE), Nankai University, Tianjin 300350, China.

[b.] Inorganic Chemistry and Catalysis Group, Debye Institute for Nanomaterials Science, Utrecht University, Universiteitsweg 99, 3584 CG Utrecht, The Netherlands. E-mail: b.m.weckhuysen@uu.nl

1 Experimental Section

1.1. Catalyst Synthesis

The parent zeolite NH_4^+-ZSM-5 was purchased from Zeolyst (CBV 2314, Lot 2200-89), with a specified Si/Al ratio of 11.5. Zeolite ZSM-5 in its proton form was obtained by static calcination in air at 550 °C before ion-exchange with Cu(II) acetate. 1 g H-ZSM-5 was suspended in 250 mL 10 mM Cu(II) acetate (Sigma-Aldrich, ≥98%) solution and stirred at room temperature for 24 h, followed by washing with a large excess of demineralized water. After drying, the zeolite was then calcined in air at 550 °C to remove the residue salt and was labelled as Cu-ZSM-5-fresh. The above-mentioned calcination procedures are identical. The zeolites were dehydrated at 150 °C for 1 h before 550 °C calcination for 4 h with a ramping rate of 2 °C/min in both heating and cooling process. The steaming procedure was modified based on our previous research[1]. The 10 % steam in air flow was produced by using a H_2O bubbler at 47 °C and was directed to the fresh zeolite Cu-ZSM-5 in a porcelain boat-shaped crucible in a tube furnace at 120 °C to prevent H_2O condensation. The steaming procedure was conducted at different temperature (650, 700, 750 or 850 °C) using a ramping rate of 2°C/min and dwell time of 16 h. The steamed Cu-exchanged zeolites ZSM-5 were then cooled down to room temperature in dry air.

1.2. Catalyst Characterization

Inductively Coupled Plasma-Optical Emission Spectroscopy (ICP-OES) was measured using a PerkinElmer Avio® 500 ICP-OES instrument at GeoLab, Utrecht University. Fresh/steamed Cu-exchanged zeolites ZSM-5 were treated with lithium borate fusion. The lithium borate fusion bead was dissolved in a 2N HNO_3 solution for ICP-OES measurement. A comparable level of Cu content (2.4 wt %) was determined in the fresh and steamed zeolites Cu-ZSM-5.

X-ray Diffraction (XRD) patterns of the fresh/steamed Cu-exchanged ZSM-5 zeolite materials were collected by a Bruker D2 Phaser with a cobalt radiation X-ray source (Co k_α = 1.789 Å). Powdered samples were pressed on the sample holder and rotated at 15 revolutions/min during measurement.

Transmission Electron Microscopy (TEM) was performed in a JEM-2800 microscope (JEOL) and operated under 200 keV. Powder samples were dispersed in ethanol and then deposited on gold TEM grids with holey carbon film.

Ammonia Temperature-Programmed Desorption (NH_3-TPD) was taken on Micromeritics Autochem II 2920 equipped with a Thermal Conductivity Detector (TCD). Typically, 60 mg fresh/steamed zeolite Cu-ZSM-5 was first dehydrated in He flow for 1 h at 600 °C with a heating ramp of 10 °C/min. Ammonia then flowed pass the sample at 100 °C, followed by flushing with He at 100 °C for 2 h. The TCD signal of ammonia desorption was recorded starting from 150 °C under a temperature ramping rate of 5°C min^{-1} in a 25 mL min^{-1} He flow and staying at 600 °C for 30 min.

Hydrogen Temperature-Programmed Reduction (H_2-TPR) was performed on Micromeritics Autochem II 2920 equipped with a TCD. Typically, 100 mg fresh/steamed zeolite Cu-ZSM-5 was reduced in 5 vol% H_2/Ar with the heating ramp of 10 °C/min. The TPR profile was recorded from 100 to 900 °C.

CO adsorbed Fourier-Transform Infrared (CO-FT-IR) spectroscopy was carried out on a Perkin Elmer 2000 FT-IR spectrometer equipped with a homemade transmission cell with CaF_2 window. A 10 mg sample was used to make the self-supported pellet for both catalysts. For each experiment, the self-supported pellet was dehydrated at 300 C for 2 h under vacuum of 10^{-6} mbar, followed by cooling down with liquid nitrogen. The reference spectrum was taken before dosing CO. The 10% CO/He (Linde, 99.998 %) was dosed in gradience to attain different surface coverage of CO. Spectra were recorded after CO dosage each time in a resolution of 4 cm^{-1} with 16 replications.

1.3. Catalyst Testing

Catalyst testing was performed in a fixed bed plug flow setup. All the required gases were provided by Linde and the flow rates of feed gases were controlled by Brooks flow meters. Typically, 50 mg sieved catalysts powders (0.125-0.425 mm) were closely packed in a quartz reactor. Prior to the NH_3-SCR reaction, NH_3 oxidation or NO oxidation reaction, the packed zeolite was activated in 5 % O_2/He at 550 °C for 1 h and was cooled to 150 °C to start the reaction.

For the standard NH_3-SCR reaction, the catalyst was exposed to SCR feed composition of 1000 ppm NO, 1000 ppm NH_3 and 5% O_2 balanced by He with a Gas Hourly Space Velocity (GHSV) of 100,000 h^{-1}. The reaction was conducted in a desired temperature from 150 to 450 °C and was stabilized for 1 hour at each target temperature. The gas composition from outlet was determined by a FT-IR gas analyzer (Perkin–Elmer, Spectrum Two) that the real-time concentration of reactants (i.e., NO

and NH_3) and products (NO_2 and N_2O) could be recorded. After the reaction reach the steady state, the average concentration of outlet gas composition was used to determine conversion and yield at each reaction temperature. The production of N_2 was calculated based on N balance assuming that only gaseous reactants/products (NO_2, NO, N_2O, N_2 and NH_3) were formed in the reaction.

$$NO\ conversion\ (\%) = \frac{[NO_{in}] - [NO_{out}]}{[NO_{in}]} \times 100\ \%$$

$$NH_3\ conversion\ (\%) = \frac{[NH_{3\ in}] - [NH_{3\ out}]}{[NH_{3\ in}]} \times 100\ \%$$

$$Yield\ (\%) = \frac{[Product]}{[NO_{in}]} \times 100\ \%$$

For a better understanding of catalytic behavior of NH_3-SCR from the sense of side reactions, ammonia oxidation reaction and nitric oxide reaction were also performed over zeolite Cu-ZSM-5-fresh and Cu-ZSM-5-850stm. The reaction processes and the conversion calculations of NH_3 oxidation as well as NO oxidation were the same as the NH_3-SCR reaction except for the compositions of gas feed. The gas feed composition was 1000 ppm NH_3 and 5% O_2 balanced by He for the NH_3 oxidation, and 1000 ppm NO and 5% O_2 balanced by He for the NO oxidation with a Gas Hourly Space Velocity (GHSV) of 100,000 h^{-1}.

1.4. *Operando* Spectroscopies

Operando UV-Vis Diffuse Reflectance Spectroscopy (DRS) was also performed during the catalyst testing and achieved by the specially designed quartz fixed-bed reactor with a UV-Vis transparent window. A high-temperature UV-vis optical fiber probe connected to an AvaSpec 2048L spectrometer was employed to collect the UV-Vis DRS spectra every 2 min.

Operando Diffuse Reflectance Infrared Fourier transform Spectroscopy (DRIFTS) was performed making use of 10-20 mg catalyst, which was loaded in a sample holder in a high temperature Harrick reaction chamber with ZnSe windows and was pressed into a pellet with flat surface. The reactant gases were flowed through the pellet from bottom to top. Samples were calcined in 5 % O_2/He at 550 °C before the reaction. The gas feed composition and reaction procedure were the same as the catalytic test on a fix bed reactor when performing the NH_3-SCR reaction. NO or NH_3 cutoff experiment was also performed at 250 °C. The DRIFTS experiment protocol is briefly illustrated in Scheme S1. The DRIFTS data were collected continuously by a Bruker Tensor II spectrometer with a MCT detector with 32 accumulation times and resolution of 4 cm^{-1}. The effluent gas composition was determined by a FT-IR gas analyzer (Perkin–Elmer, Spectrum Two).

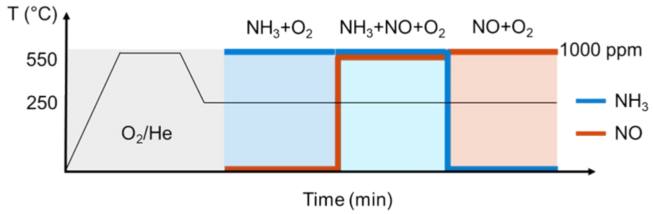

Scheme S1. Procedure of *operando* Diffuse Reflectance Infrared Fourier Transform Spectrsocopy (DRIFTS) experiments at 250 °C, with 1000 ppm of NH_3 and/or 1000 ppm NO balanced by 5% O_2/He.Gases flowed in the sequence of NH_3+O_2, NH_3+NO+O_2 and NO+O_2.

2 Additional Results

2.1 Catalyst Testing

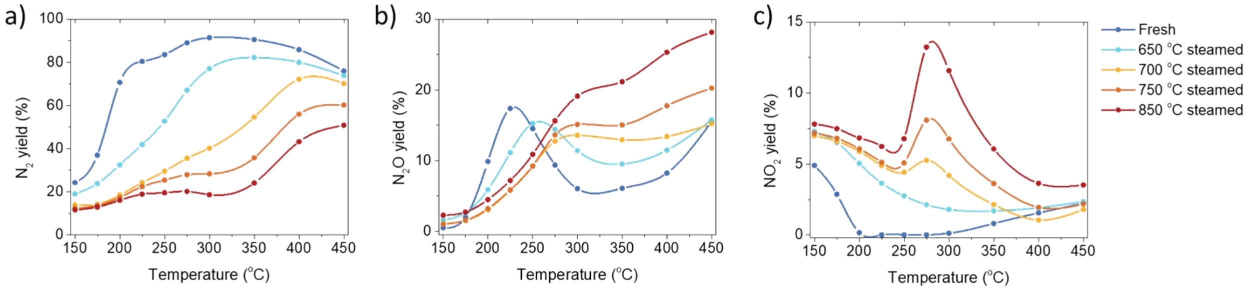

Figure S1. Standard NH_3-Selective Catalytic Reduction (SCR) of NO reaction performance of a fresh and steamed Cu-exchanged ZSM-5: a) N_2 yield, b) N_2O yield and c) NO_2 yield. The reaction was conducted at a Gas Hourly Space Velocity (GHSV) of 100,000 h^{-1} with 1000 ppm NO, 1000 ppm NH_3, 5% O_2 and balanced with He.

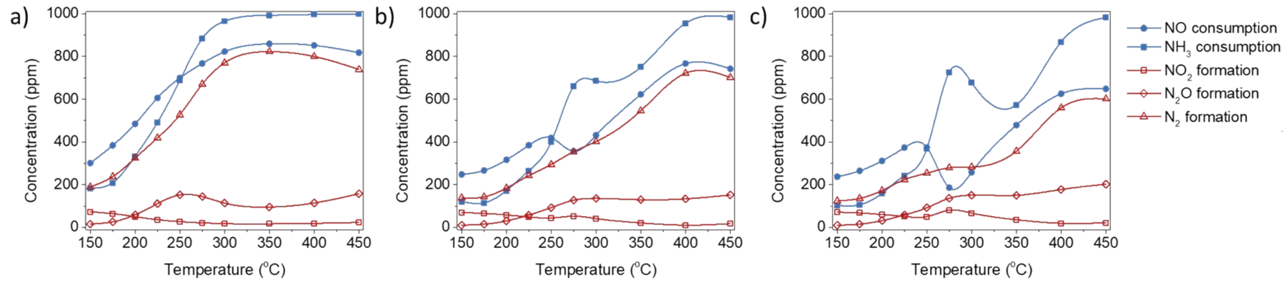

Figure S2. The reactants consumption and products formation on a a) 650 °C, b) 700 °C and c) 750 °C steamed zeolite Cu-ZSM-5 in the standard NH_3-Selective Catalytic Reduction (SCR) of NO reaction. The reaction was conducted at a Gas Hourly Space Velocity (GHSV) of 100,000 h^{-1} with 1000 ppm NO, 1000 ppm NH_3, 5% O_2 and balanced with He.

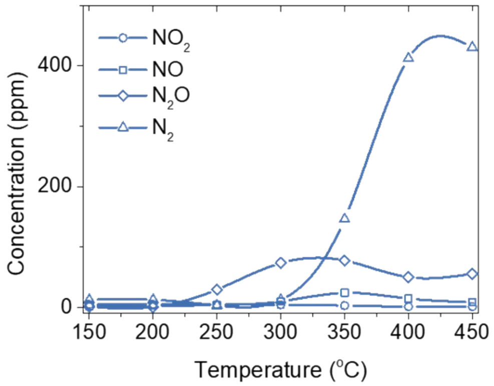

Figure S3. Product concentration from NH_3 oxidation performed on fresh zeolite Cu-ZSM-5. The reaction was conducted at a Gas Hourly Space Velocity (GHSV) of 100,000 h^{-1} with 1000 ppm NH_3, 5% O_2 and balanced with He.

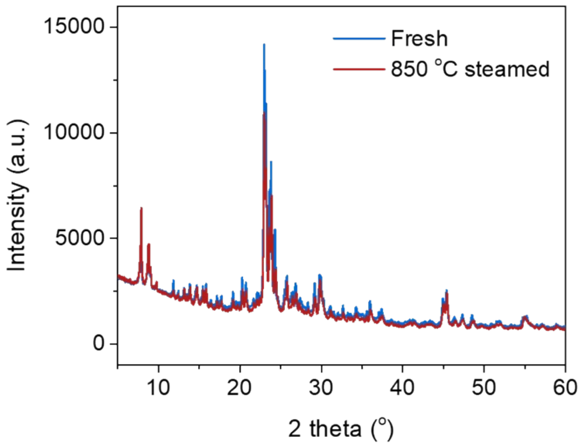

2.2 Catalyst Characterization

Figure S4. X-ray Diffraction (XRD) patterns of the fresh and 850 °C steamed zeolite Cu-ZSM-5.

Figure S5. Fourier Transform-Infrared (FT-IR) data of the OH stretching region of the fresh and 850 °C steamed zeolite Cu-ZSM-5, recorded before cooling down to liquid N_2 temperature for CO dosage in CO-FT-IR spectroscopy experiment.

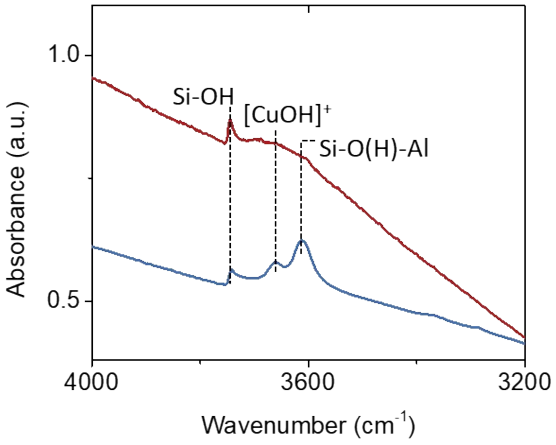

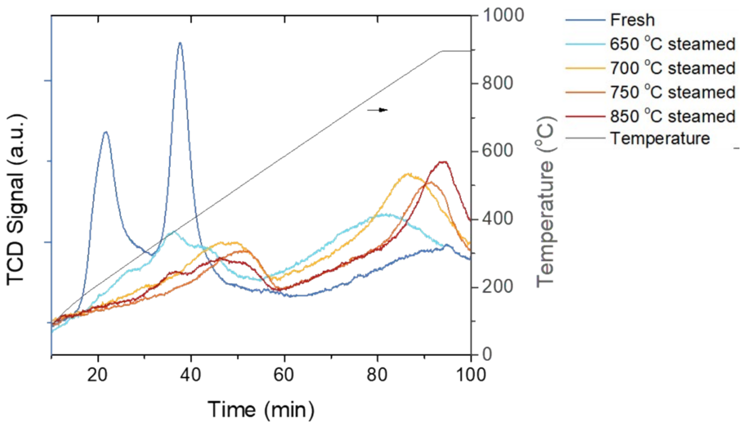

Figure S6. H_2-Temperature Programmed Reduction (TPR) profiles of the fresh and steamed zeolites Cu-ZSM-5. The Cu^{2+} ions in fresh Cu-ZSM-5 were reduced to isolated Cu^{+} at around 200 °C followed by the consecutive reduction at 400 °C. Whereas in the steamed zeolite, full reduction of Cu^{2+} to metallic Cu took place above 600 °C, probably due to the strong interaction of Cu with extra-framework Al causing the formation of a $CuAl_xO_y$ phase or the stabilization of monovalent Cu^{+} in the highly negatively charged zeolite framework.[2]

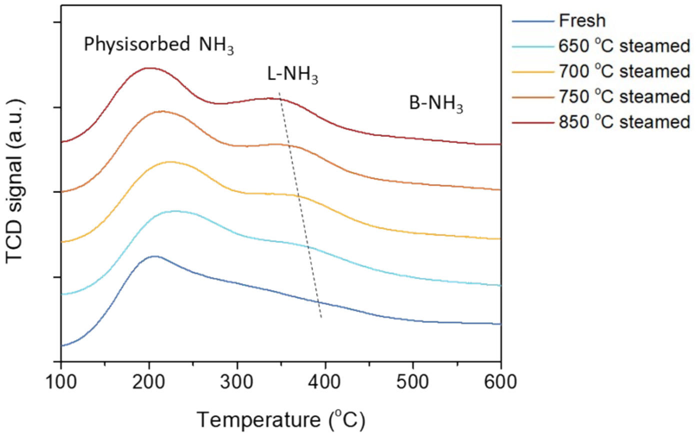

Figure S7. NH_3-Temperature Programmed Desorption (TPD) profiles of the fresh and steamed zeolites Cu-ZSM-5.

2.3 *Operando* UV-Vis Diffuse Reflectance Spectroscopy Experiments

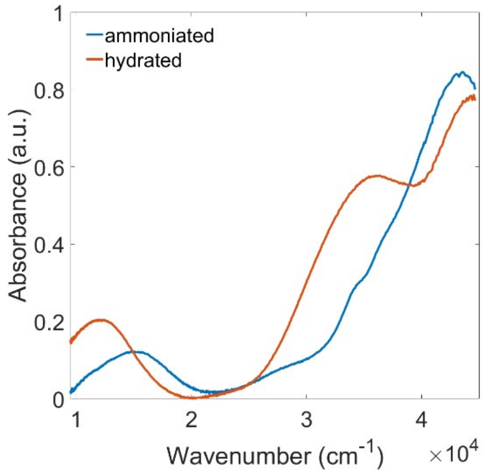

Figure S8. UV-Vis Diffuse Reflectance Spectroscopy (DRS) data of hydrated and ammoniated Cu^{2+} in the fresh Cu-ZSM-5. The spectrum of ammoniated zeolite was obtained under NH_3/He flow after dehydration.

Figure S8 shows the UV-Vis diffuse reflectance spectroscopy (DRS) data of hydrated and ammoniated Cu^{2+} in the fresh zeolite Cu-ZSM-5. The hybridized metal-ligand complex orbital affects the splitting of the d orbital of the metal. As a result, varying the type of the ligand gives rise to different features in both the d-d transition and the LMCT region in a UV-Vis spectrum; the position shift in the Ligand-to-Metal Charge Transfer (LMCT) transition is usually more significant and noticeable since the energy difference between orbitals with ligand and metal character is larger than that between metal orbitals. Such charge transfer transition reflects the Lewis basicity or electron donor properties of the coordinated ligands, which is expressed in terms of optical electronegativity and gives the measure of covalency between metal and ligands reflected in the position of the LMCT band.[3]

The d-d transition bands with the maximum at a 12200 cm^{-1} and 15500 cm^{-1} are typical features of $[Cu(H_2O)_6]^{2+}$ and $[Cu(NH_3)_4(H_2O)_x]^{2+}$ (x = 1, 2) complexes, respectively.[4] The constraint from the zeolite framework might cause the tetragonal distortion of the Cu complex, which adjusts the d orbital splitting of Cu^{2+} and consequently leads to a blue shift of the band position.[5] The ligand field generated by NH_3 is stronger than H_2O, providing only σ-donation from the ligand bonding orbital. The σ-donor ensures a strong metal-ligand overlap that results in an increased level of antibonding orbital, *i.e.*, a larger value of the splitting parameter, which further caused the blue shift of the d-d transition band.[6]

The LMCT band usually presents as a broad and intense band as the charge transfer is fully allowed between metal and ligand.[7] The LMCT band lying above 30000 cm^{-1} in the hydrated Cu-ZSM-5 originated from a O^{2-} to Cu^{2+} charge transfer, which superimposes on the fundamental absorption edge of the zeolite matrix.[8,9] The LMCT band of ammoniated Cu^{2+} is located at a higher wavenumber of around 42000 cm^{-1} along with a shoulder at around 32000 cm^{-1}, suggesting the existence of one or more H_2O ligands in the ammoniated Cu^{2+} complex.

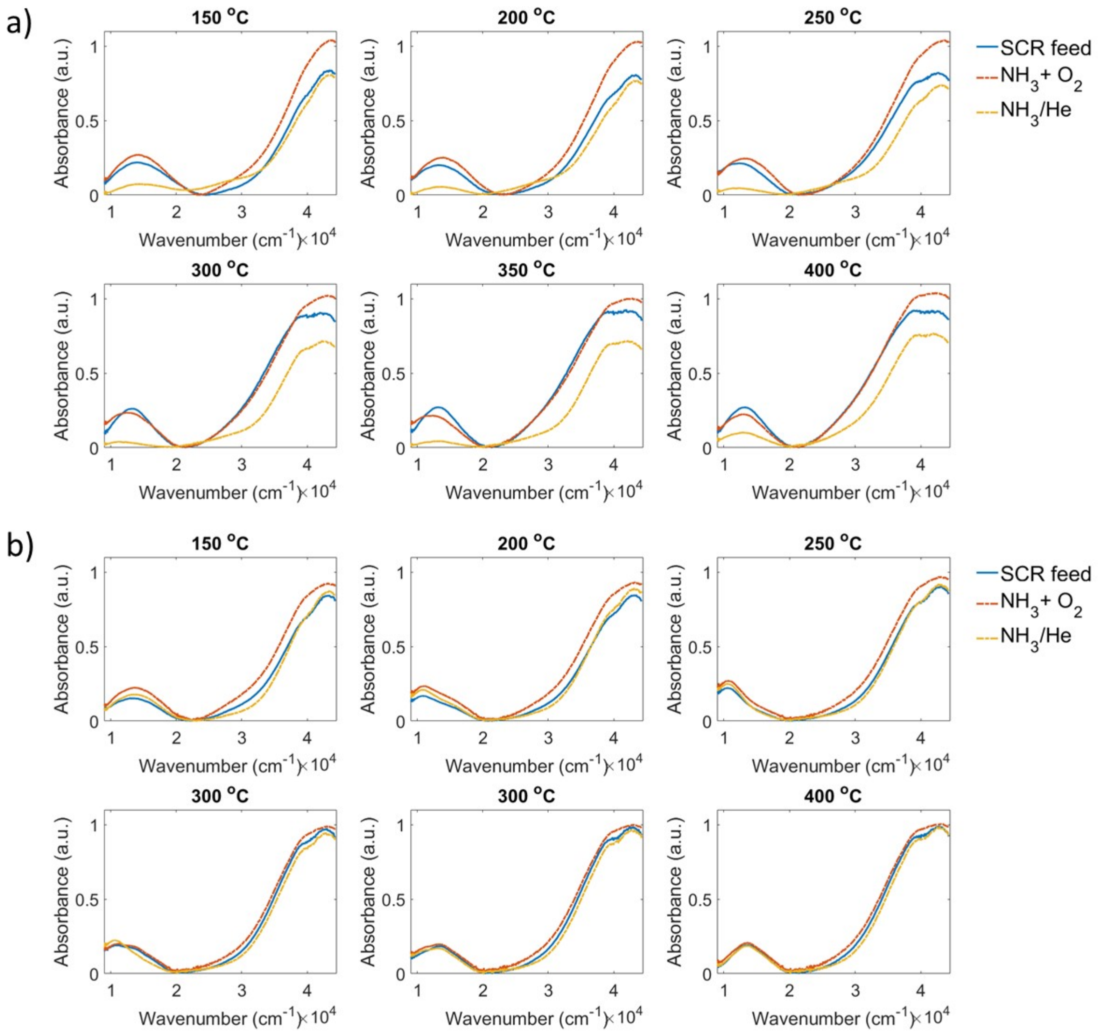

Figure S9. UV-Vis Diffuse Reflectance Spectroscopy (DRS) data of the a) fresh and b) 850 °C steamed zeolite Cu-ZSM-5 in standard NH_3-Selective Catalytic Reduction (SCR) reaction of NO (blue), NH_3 oxidation reaction (red) as well as NH_3/He (yellow) flow at different reaction temperatures of 150-400 °C.

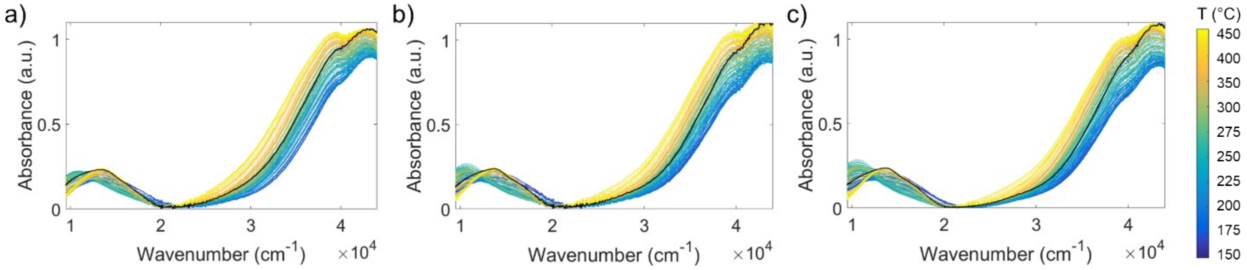

Figure S10. *Operando* UV-Vis Diffuse Reflectance Spectroscopy (DRS) data of the a) 650 °C, b) 700 °C and c) 750 °C steamed zeolite Cu-ZSM-5 during the NH_3-Selective Catalytic Reduction (SCR) reaction of NO.

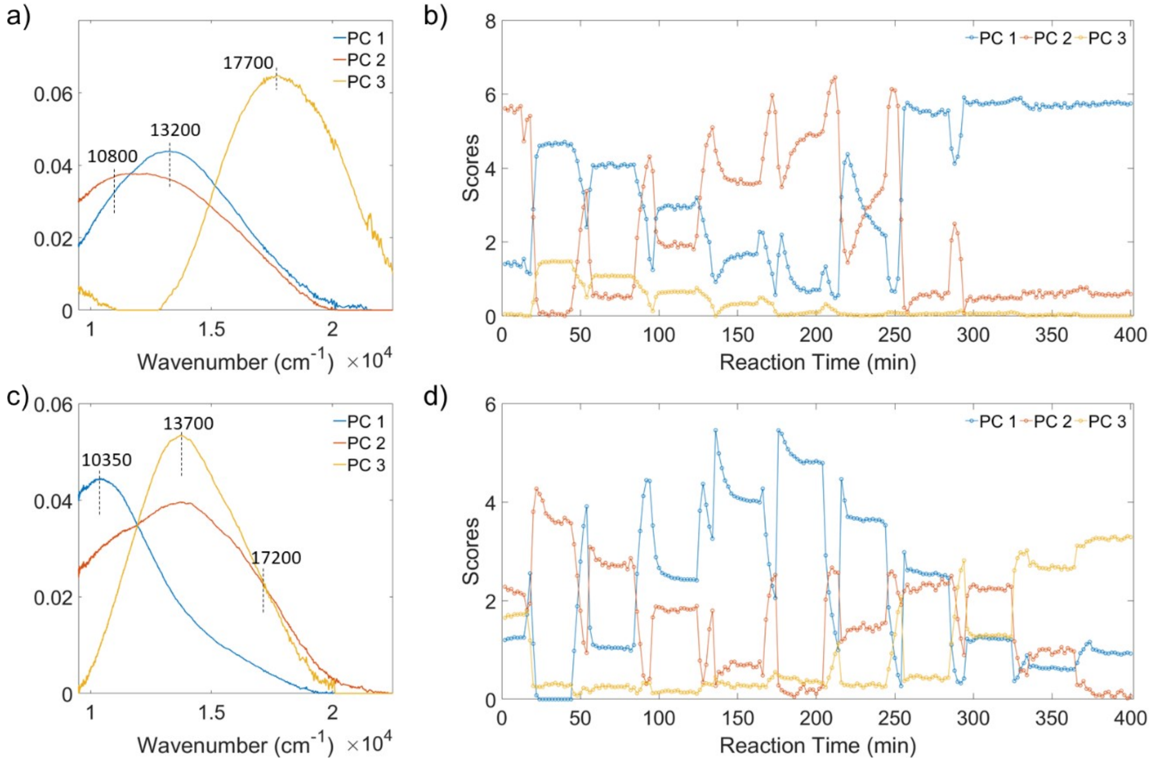

Figure S11. Principal Component Analysis (PCA) results of the *operando* UV-Vis Diffuse Reflectance Spectroscopy (DRS) data of the a-b) fresh and c-d) 850 °C steamed zeolites Cu-ZSM-5. The first three principal components separate well the low, medium, and high wavenumber bands, from where the band positions can be determined accurately for the subsequent Gaussian fitting of each spectrum of the series. The scores indicate the contributions of the three different components in each spectrum of the series.

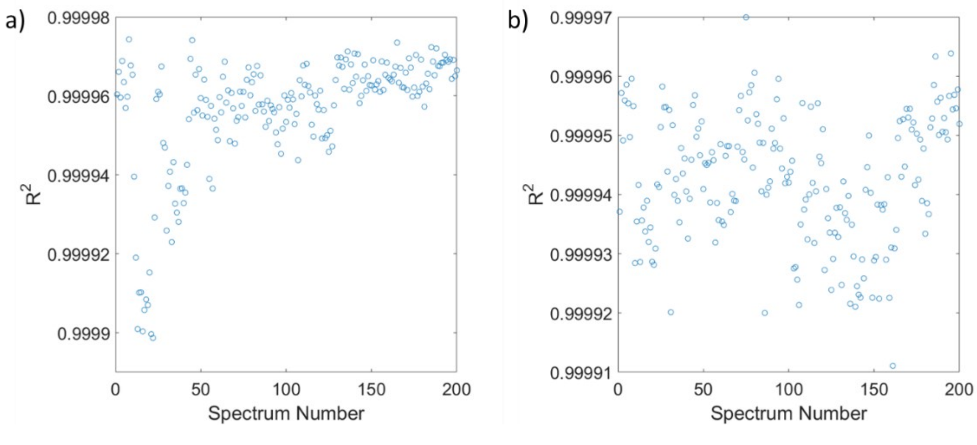

Figure S12. Goodness of fit in terms of R square of the fitted d-d transition band in all fitted spectra obtained during NH_3 Selective Catalytic Reduction (SCR) reaction of NO performed on the a) fresh and b) 850 °C steamed zeolite Cu-ZSM-5.

The d-d transition band of Cu^{2+} was fitted using Gaussian functions. By inspecting the evolution of the d-d transition band under reaction condition via Principal Component Analysis (PCA), the d-d transition band was fitted using three Gaussian peaks, whose peak positions were restrained to the regions 10200-10800 cm^{-1}, 13000-14000 cm^{-1}, and 16000-18000 cm^{-1}. In addition, the peak width was confined as well based on the initial fitting result of a reference spectrum (UV-Vis diffuse reflectance spectrum of fresh zeolite Cu-ZSM-5 in He at 150 °C). All spectra were fitted using the same fitting parameters. The R square of fit reached the level of 0.9999 for every fitted spectrum. The fitting curves and individual fitted components of fresh and 850 °C steamed zeolite Cu-ZSM-5 are respectively shown in Figures S13-14 to indicate the reliability of the fitting.

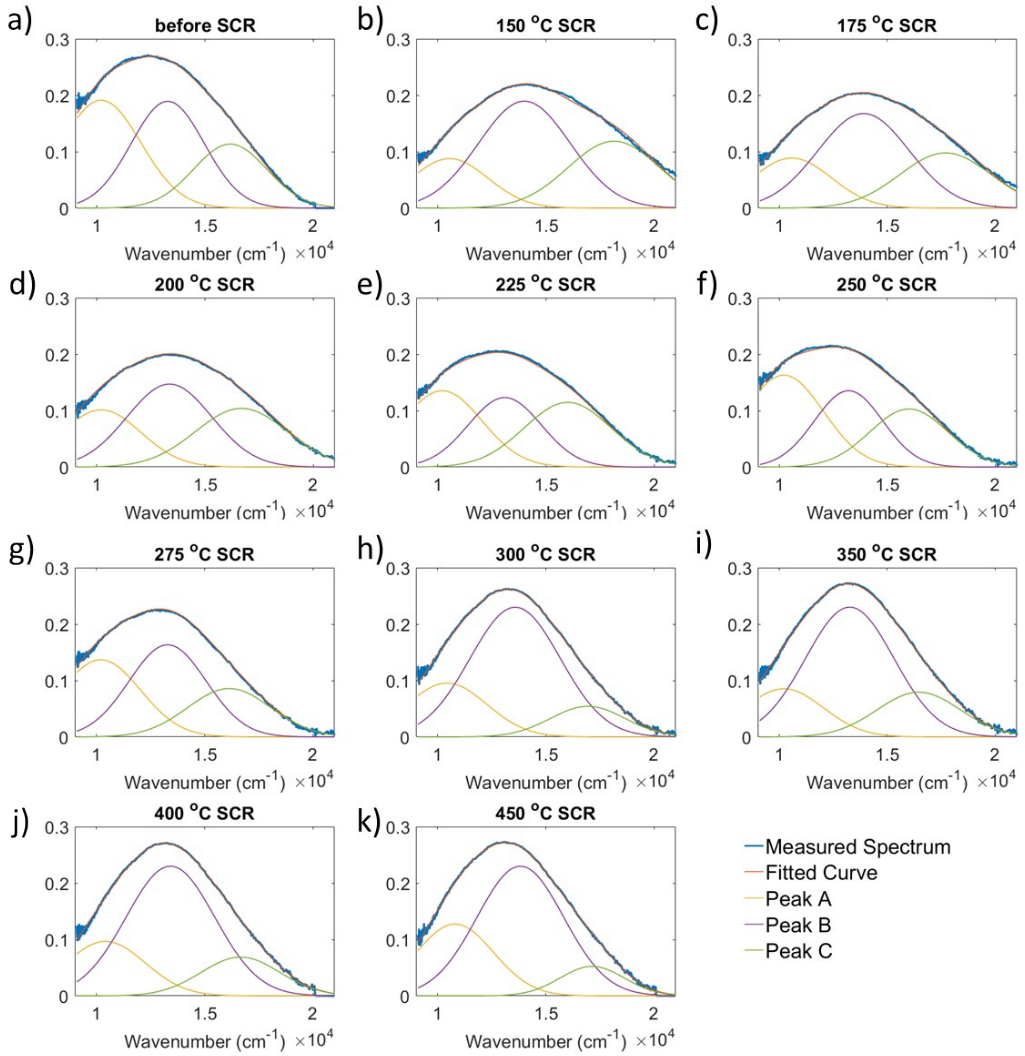

Figure S13. Fitting curves and the corresponding bands of the fitting model in the d-d transition region in a UV-Vis diffuse reflectance spectrum before standard NH_3-Selective Catalytic Reduction (SCR) reaction of NO (a) and under steady-state conditions at different reaction temperatures (b-k) performed on the fresh zeolite Cu-ZSM-5 material.

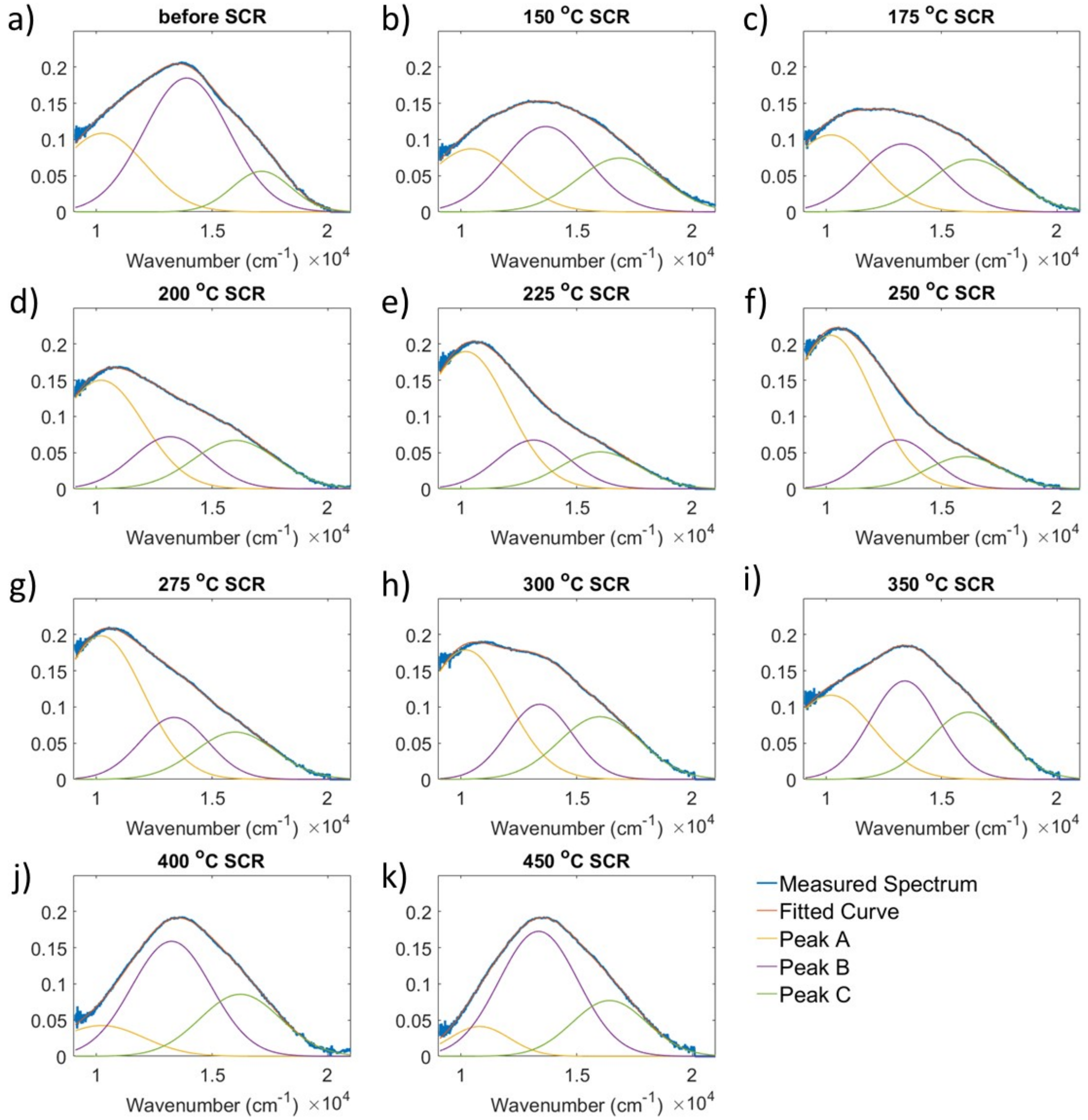

Figure S14. Fitting curves and the corresponding bands of the fitting model in d-d transition region in a UV-Vis diffuse reflectance spectrum before standard NH_3-Selective Catalytic Reduction (SCR) reaction of NO (a) and under steady-state conditions at different reaction temperature (b-k) performed on 850 °C steamed zeolite Cu-ZSM-5 material.

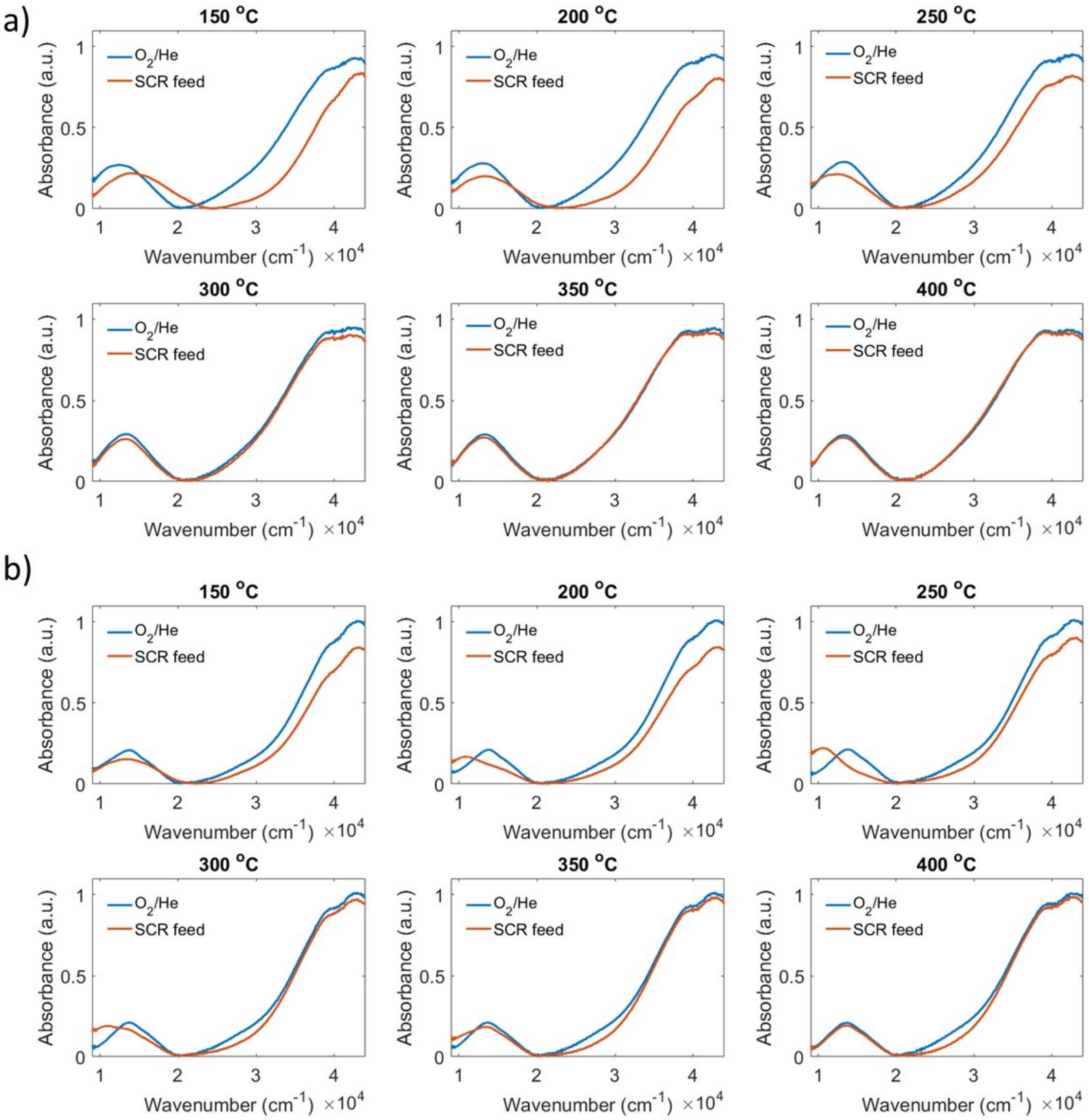

Figure S15. UV-Vis diffuse reflectance spectra of the a) fresh and b) 850 °C steamed zeolite Cu-ZSM-5 obtained after calcination during the cooling procedure in O_2/He (blue), and under Selective Catalytic Reduction (SCR) feed (red) at different reaction temperatures between 150-400 °C.

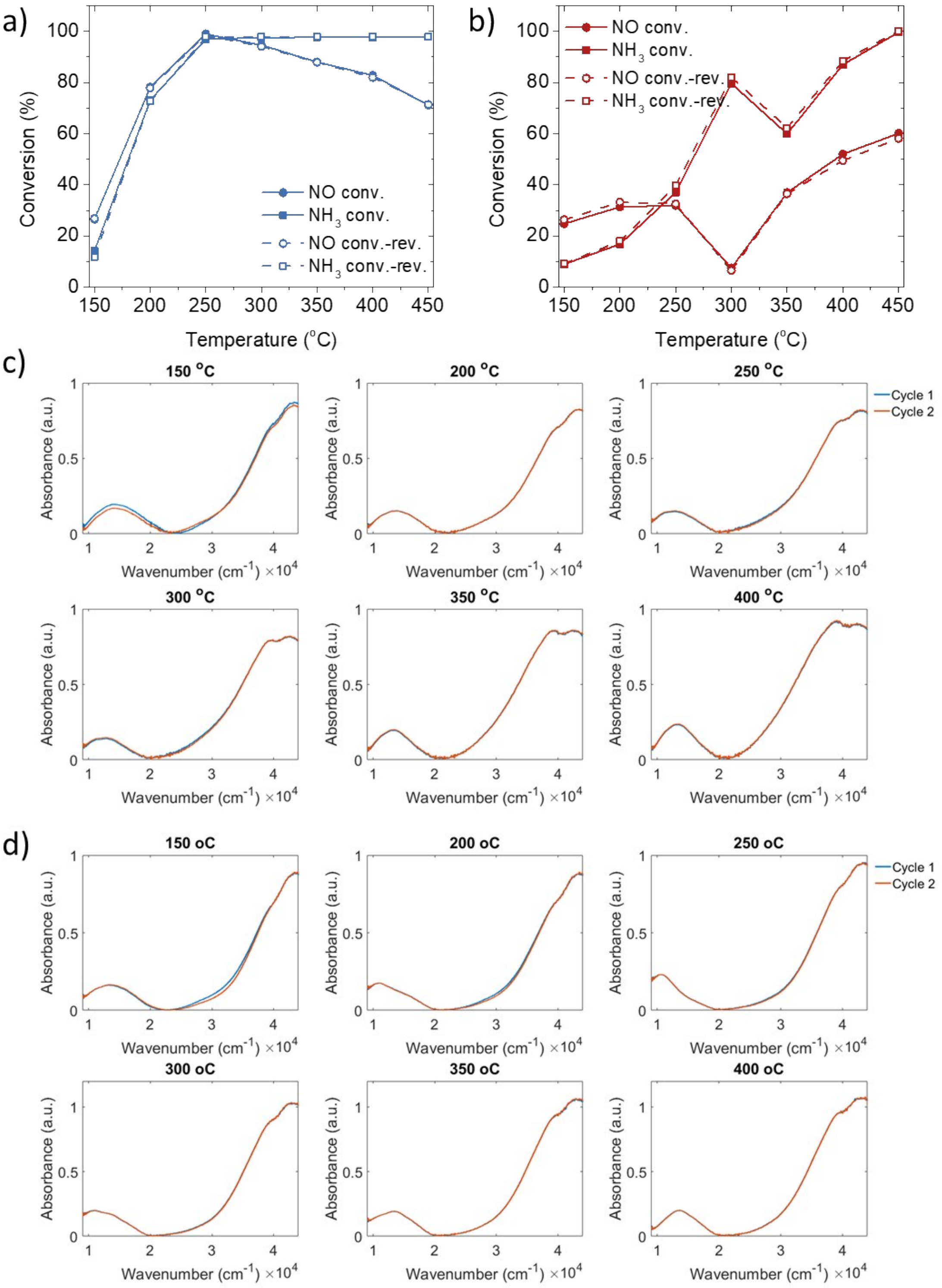

Figure S16. NO and NH_3 conversion of the NH_3-Selective Catalytic Reduction (SCR) of NO (in the temperature window of 150-450 °C) and its reversed cycle (450-150 °C) over the a) fresh and b) 850 °C steamed zeolites Cu-ZSM-5. The corresponding *operando* UV-Vis diffuse reflectance spectra of the c) fresh and d) 850 °C steamed zeolites Cu-ZSM-5 were also collected in the reaction, and cycle 2 represent the reversed cycle. The comparable catalytic performance and spectroscopic observations in the two NH_3-SCR cycles strongly suggest that the evolution of species observed during the first cycle was reversible, and not due to irreversible deactivation and degradation of Cu species (which may nonetheless occur over multiple catalytic cycles or prolonged testing).

2.4 *Operando* Diffuse Reflectance Infrared Fourier Transform Spectroscopy Experiments

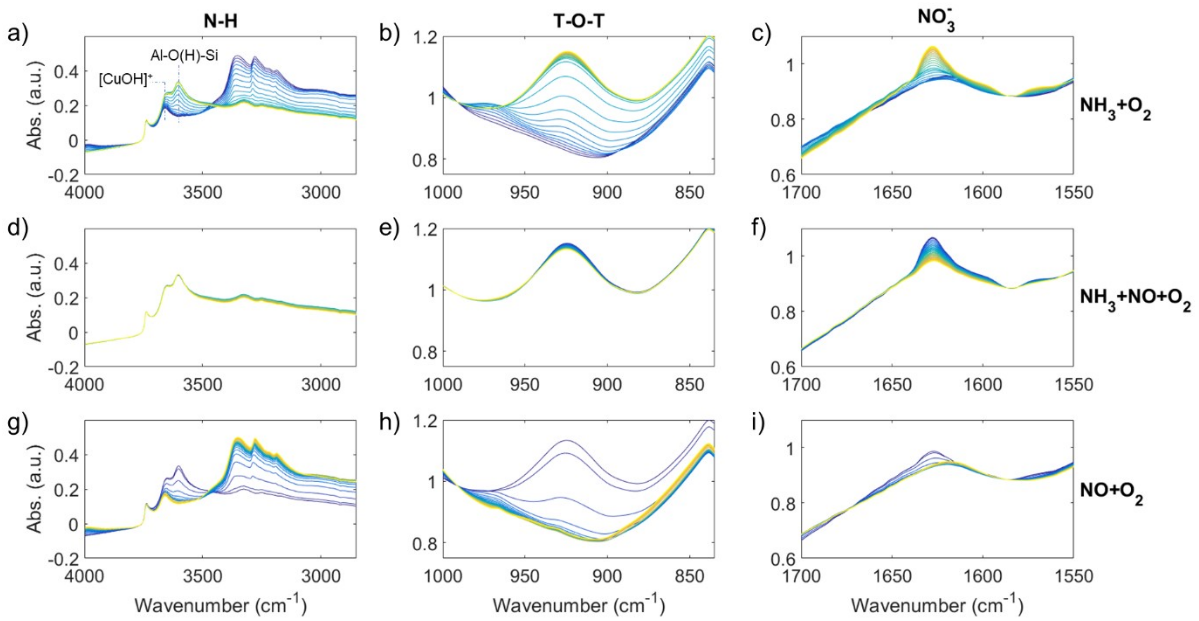

Figure S17. *Operando* Diffuse Reflectance Infrared Fourier Transform Spectroscopy (DRIFTS) data of fresh zeolite Cu-ZSM-5 maintained recorded in the flow of (a-c) NH_3+O_2, (d-f) NH_3+NO+O_2 and (g-i) NO+O_2 at 250 °C for 30 min. Reactant gases were balanced by He. The experimental procedure can be found in Scheme S1. The vibrational regions of O-H/N-H stretching (a, d, g), perturbed framework T-O-T vibration (b, e, h) and N=O stretching (c, f, i) are shown. The color range indicates spectral changes from blue to yellow following the reaction time in each subfigure.

The stretching vibration of O-H in the hydroxyl group in a Cu-exchanged zeolite is well documented.[10] The absorption band at 3738 cm^{-1}, 3659 cm^{-1} as well as 3602 cm^{-1} is assigned to O-H vibration in external silanol, $[CuOH]^+$ and Brønsted acid respectively. The stronger acidity presents a lower frequency due to a more ready-to-donate proton and thus a longer O-H bond. The N-H bond of adsorbed NH_3 has a lower stretching vibrational frequency than that of a hydroxyl group in zeolites. NH_3 is chemisorbed on the catalyst surface via the lone electron pair from the N side of NH_3. A few N-H bands are observed in 3400-3100 cm^{-1} region, which are typical N-H asymmetric and symmetric stretching $v_s(NH_3)$ modes as well as the splitting of $v_s(NH_3)$ due to a Fermi resonance with the overtone of asymmetric NH_3 deformation.[11,12] It is noted that upon the consumption of the NH_3 bands the $[CuOH]^+$ developed first, followed by the depletion of Brønsted acid in the NH_3 oxidation. The interaction of NH_3 with the Brønsted acid site generates NH_4^+ that could be tracked by the N-H bending vibration at ~1460 cm^{-1}. Finally, the broad feature at around 3000 cm^{-1} indicates the formation of H-bonded NH_3 with a surface hydroxyl group.

When the NH_3-SCR gases were fed, Cu cations solvated with NH_3 to form the stable ammoniated-Cu^{2+}, which is mobile and allowed to free the Cu ions from their charge balanced positions. The elimination of this Cu-framework interaction allowed the framework T-O-T network to relax to the original vibration. Therefore, the perturbed framework vibrational band vanished along with the NH_3 adsorption (Figure S17-20). By exposure to SCR gas composite, the Cu moieties interacted with reactants such as O_2, or the intermediates such as NO_x^-, which caused the slight shift of the bands.

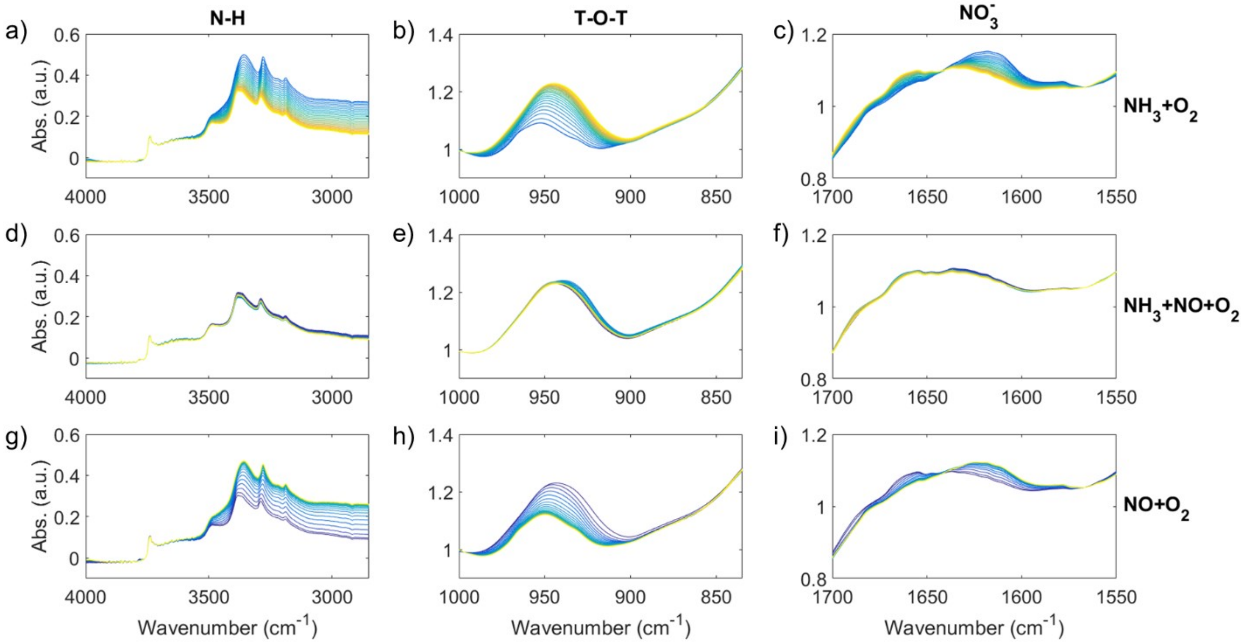

Figure S18. *Operando* Diffuse Reflectance Infrared Fourier Transform Spectroscopy (DRIFTS) data of an 850 °C steamed zeolite Cu-ZSM-5 recorded during a flow of (a-c) NH_3+O_2, (d-f) NH_3+NO+O_2 and (g-i) NO+O_2 at 250 °C for 30 min. Reactant gases were balanced by Helium. The experimental procedure can be found in Scheme S1. The vibrational regions of O-H/N-H stretching (a, d, g), perturbed framework T-O-T vibration (b, e, h), and N=O stretching (c, f, i) are shown. The colors indicate spectral changes from blue to yellow following the reaction time in each subfigure.

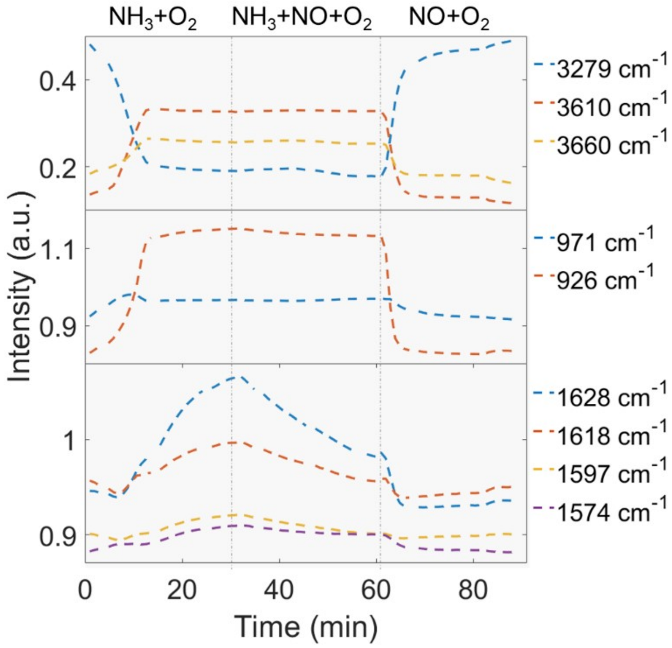

Figure S19. Evolution of the Diffuse Reflectance Infrared Fourier Transform Spectroscopy (DRIFTS) absorption bands representing adsorbed NH_3 (upper panel), perturbed framework T-O-T vibration (middle panel), and surface nitrates (bottom panel) as followed for the fresh zeolite Cu-ZSM-5 during the *operando* DRIFTS experiment. The experimental procedure can be found in Scheme S1.

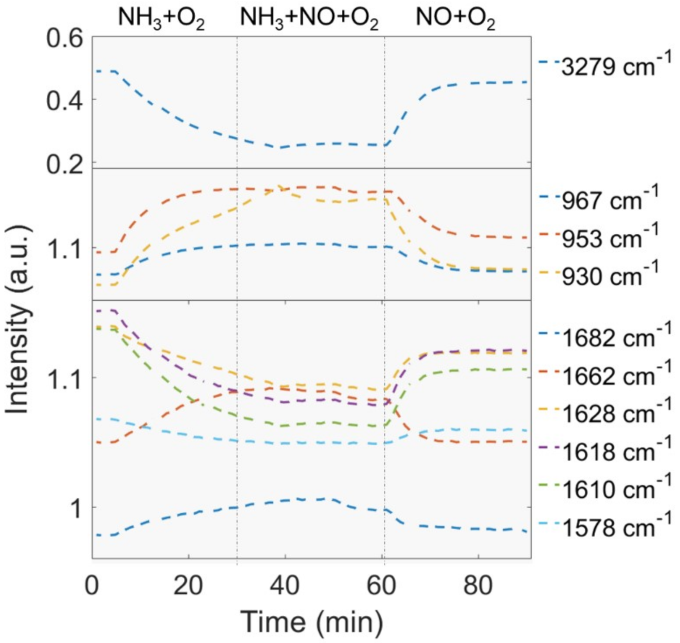

Figure S20. Evolution of the Diffuse Reflectance Infrared Fourier Transform Spectroscopy (DRIFTS) absorption bands representing adsorbed NH_3 (upper panel), perturbed framework T-O-T vibration (middle panel), and surface nitrates (bottom panel) as followed for the 850 °C steamed zeolite Cu-ZSM-5 during the *operando* DRIFTS experiment. The experimental procedure can be found in Scheme S1.

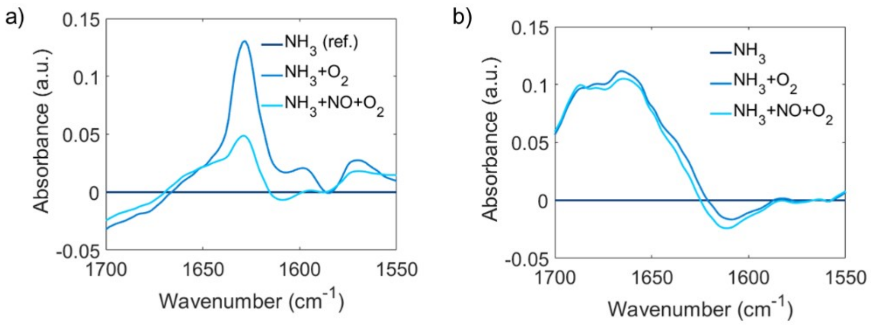

Figure S21. The difference spectra of a) fresh and b) 850 °C steamed Cu-ZSM-5 taking spectrum in NH_3 flow as reference spectrum.

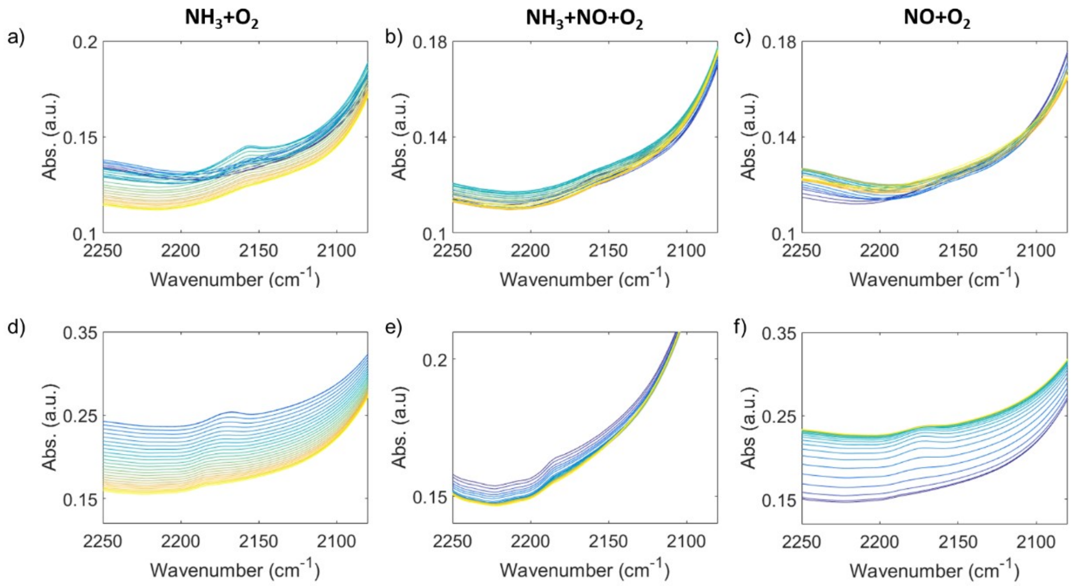

Figure S22. *Operando* Diffuse Reflectance Infrared Fourier Transform Spectroscopy (DRIFTS) data showing the formation of surface nitrosonium ion NO^+ on a-c) fresh and d-f) 850 °C steamed zeolite Cu-ZSM-5 recorded during a flow of NH_3+O_2, NH_3+NO+O_2 and NO+O_2 at 250 °C for 30 min. Reactant gases were balanced by He. The experimental procedure can be found in Scheme S1. The colors indicate spectral changes from blue to yellow following the reaction time in each subfigure.

3 Additional References

1 X. Ye, J. E. Schmidt, R. Wang, I. K. Ravenhorst, R. Oord, T. Chen, F. Groot, F. Meirer and B. M. Weckhuysen, *Angew. Chem. Int. Ed.*, 2020, **59**, 15610–15617.

2 R. Bulánek, B. Wichterlová, Z. Sobalík and J. Tichý, *Appl. Catal. B Environ.*, 2001, **31**, 13–25.

3 R. A. Schoonheydt, *Chem. Soc. Rev.*, 2010, **39**, 5051–5066.

4 A. Delabie, K. Pierloot, M. H. Groothaert, B. M. Weckhuysen and R. A. Schoonheydt, *Microporous Mesoporous Mater.*, 2000, **37**, 209–222.

5 W. De Wilde, R. A. Schoonheydt and J. B. Uytterhoeven, in *ACS Symposium Series*, 1977, pp. 132–143.

6 P. Atkins, T. Overton, J. Rourker and M. Weller, *Shriver and Atkin’s Inorganic Chemistry, Fifth Edition*, Oxford University Press, Oxford, 2010.

7 F. Giordanino, P. N. R. Vennestrøm, L. F. Lundegaard, F. N. Stappen, S. Mossin, P. Beato, S. Bordiga and C. Lamberti, *Dalt. Trans.*, 2013, **42**, 12741–12761.

8 A. A. Verma, S. A. Bates, T. Anggara, C. Paolucci, A. A. Parekh, K. Kamasamudram, A. Yezerets, J. T. Miller, W. N. Delgass, W. F. Schneider and F. H. Ribeiro, *J. Catal.*, 2014, **312**, 179–190.

9 M. H. Groothaert, K. Lievens, H. Leeman, B. M. Weckhuysen and R. A. Schoonheydt, *J. Catal.*, 2003, **220**, 500–512.

10 F. Giordanino, E. Borfecchia, K. A. Lomachenko, A. Lazzarini, G. Agostini, E. Gallo, A. V. Soldatov, P. Beato, S. Bordiga and C. Lamberti, *J. Phys. Chem. Lett.*, 2014, **5**, 1552–1559.

11 N. Topsoe, *J. Catal.*, 1991, **128**, 499–511.

12 G. Ramis, G. Busca, V. Lorenzelli and P. Forzatti, *Appl. Catal.*, 1990, **64**, 243–257.
